# Minimal Peptide Sequences That Undergo Liquid–Liquid Phase Separation via Self-Coacervation or Complex Coacervation with ATP

**DOI:** 10.1021/acs.biomac.4c00738

**Published:** 2024-07-27

**Authors:** Valeria Castelletto, Jani Seitsonen, Alice Pollitt, Ian W. Hamley

**Affiliations:** † School of Chemistry, Food Biosciences and Pharmacy, University of Reading, Whiteknights, Reading RG6 6AD, U.K.; ‡ Nanomicroscopy Center, Aalto University, Puumiehenkuja 2, Espoo 02150, Finland; § Institute for Cardiovascular and Metabolic Research, School of Biological Sciences, University of Reading, Reading RG6 6AS, U.K.

## Abstract

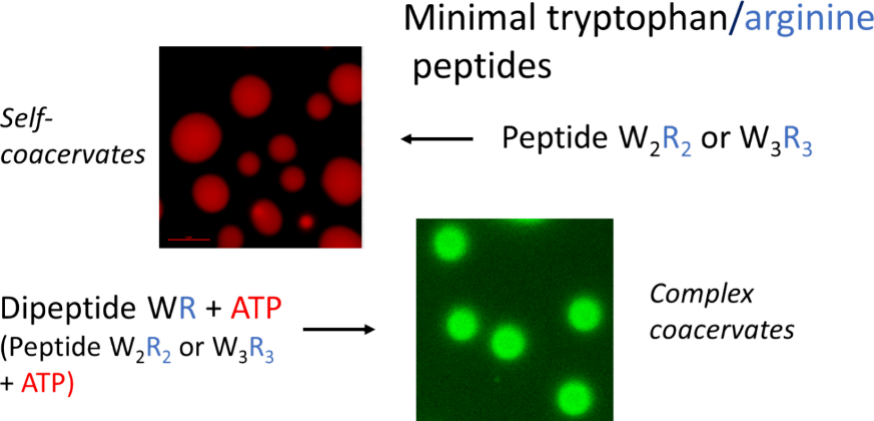

The simple (self-)coacervation of the minimal tryptophan/arginine peptide sequences W_2_R_2_ and W_3_R_3_ was observed in salt-free aqueous solution. The phase diagrams were mapped using turbidimetry and optical microscopy, and the coacervate droplets were imaged using confocal microscopy complemented by cryo-TEM to image smaller droplets. The droplet size distribution and stability were probed using dynamic light scattering, and the droplet surface potential was obtained from zeta potential measurements. SAXS was used to elucidate the structure within the coacervate droplets, and circular dichroism spectroscopy was used to probe the conformation of the peptides, a characteristic signature for cation−π interactions being present under conditions of coacervation. These observations were rationalized using a simple model for the Rayleigh stability of charged coacervate droplets, along with atomistic molecular dynamics simulations which provide insight into stabilizing π–π stacking interactions of tryptophan as well as arginine–tryptophan cation−π interactions (which modulate the charge of the tryptophan π-electron system). Remarkably, the dipeptide WR did not show simple coacervation under the conditions examined, but complex coacervation was observed in mixtures with ATP (adenosine triphosphate). The electrostatically stabilized coacervation in this case provides a minimal model for peptide/nucleotide membraneless organelle formation. These are among the simplest model peptide systems observed to date able to undergo either simple or complex coacervation and are of future interest as protocell systems.

## Introduction

Understanding liquid–liquid phase separation (LLPS) can shed light on the formation of membraneless organelles, which have roles in subcellular processes including the formation of stress granules, P granules, cell signaling or chromatin packing.^1−6^ As well as physiological roles, LLPS is also associated with pathological processes including amyloid formation responsible for neurodegenerative diseases and the regulation of cell signaling in cancer.^1,6−10^ LLPS of simple components such as short peptides and nucleotides has been proposed as a protocell model relevant to the prebiotic emergence of compartmentalized particles.^11−13^ The membraneless nature of the droplets enables easy transport across the surface and can encapsulate molecules under crowded conditions. In one example, it was shown that coacervate microdroplets are also able to encapsulate photoactive molecules, catalytic nanoparticles and enzymes, with enhanced activity compared to bulk.^11^ LLPS has been shown to play a role in certain types of biological adhesion^14,15^ and may have activity in ice crystal growth inhibition,^16^ and related applications involving molecular crowding in the continuous interstitial volume between densely packed coacervate droplets. Coacervates also have applications in the formulation of pharmaceutical, personal care, food and agricultural products.^17^ LLPS is a phase separation process that may result from simple coacervation of a single species in solution (self-coacervation) or complex coacervation between different species, generally oppositely charged polyelectrolytes. LLPS may result from associative or segregative phase separation.^18,19^ The former results from attractive interactions between molecules, the latter occurs when the components of a mixture are repelled from each other due to unfavorable intermolecular interactions. In associative LLPS, dilute and dense phases with different concentrations coexist, whereas there is considerable separation of components in segregative phase separation. Simple coacervation and complex coacervation result from associative phase separation.

Arginine has a range of important biological activities^20^ and is known to play a role in LLPS of proteins,^3,4,6,21−25^ originally ascribed to cation−π interactions between arginine and aromatic residues. However, this has been examined in detail^6,25^ and it has been shown that due to the sp^2^ nature of the delocalized amine bonds in the guanidinium group, arginine can participate in hydrophobic π–π interactions [Arg–Arg (R–R) or Arg–X where X is a hydrophobic residue, Phe, Tyr or Trp (W)], in particular at high salt concentration where electrostatic interactions are screened and hydrophobicity is increased by salt-induced entropic effects.^6^

In an effort to develop minimal peptide sequence to model LLPS in intrinsically disordered proteins (IDPs), a 14-residue peptide WGRGRGRGWPGVGY was developed along with several analogs, the shortest sequence being WGRGRGRGWY.^26^ The peptides were observed to form droplets by LLPS in the presence of salt due to simple coacervation driven by π–π interactions between R and aromatic residues (Y or F).^26^

All 400 dipeptides (based on the 20 naturally occurring amino acids) have been screened for potential to exhibit LLPS behavior^27^ (as opposed to previously studied aggregation propensity^28^) by computational methods (coarse-grained molecular dynamics). One lead candidate dipeptide QW that was identified as an LLPS candidate was studied in more detail experimentally (turbidity and optical microscopy experiments) and by simulation methods, which revealed the importance of anion−π, cation−π and hydrogen bonding interactions.^27^ A *tert*-butyl cationic FF (diphenylalanine) dipeptide undergoes LLPS.^29^ The dipeptide FF-OMe (C-terminal methoxylated diphenylalanine) also shows LLPS upon increase of pH, as do related methoxylated tripeptides FFG-OMe and FFE-OMe.^30^ In another recent example, LLPS was demonstrated for short arginine- or histidine-containing 22-residue peptides with IDP-like spacers (repeat GLG sequences).^31^

Here, we report on the development of novel minimal peptide sequences WWRR (W_2_R_2_) and WWWRRR (W_3_R_3_) that undergo LLPS (simple coacervation) in aqueous solution at sufficiently high pH, attained by titration of NaOH. The shorter peptide WR did not show self-coacervation under the conditions examined. The sequences were designed to contain arginine and the aromatic residue tryptophan to enable π–π stacking and to facilitate potential cation−π interactions. The influence of cation−π interactions on the charge distribution within aromatic residues Tyr and Phe induced by Arg has been reported via electrostatic surface potential calculations,^6^ here we probe such interactions as contributors to the simple coacervation of W_2_R_2_ and W_3_R_3_.

We then present minimal protocell mimics, in solutions containing mixtures of peptide (including dipeptide WR) and the metabolic energy source mononucleotide adenosine triphosphate (ATP). We found that ATP in the solution triggers LLPS in samples containing WR in water, while it leads to phase separation with simultaneous LLPS in samples containing W_2_R_2_ or W_3_R_3_.

## Experimental Section

### Materials

Peptides were obtained from Peptide Synthetics (Peptide Protein Research), Farnham, UK as TFA salts with >95% purity as confirmed by RP-HPLC. Molar masses by ESI-MS are as follows. WR: 360.40 g mol^–1^ (360.19 g mol^–1^ expected); W_2_R_2_: 702.80 g mol^–1^ (702.37 g mol^–1^ expected); W_3_R_3_: 1045.20 g mol^–1^ (1044.55 g mol^–1^ expected).

Adenosine 5′-triphosphate (ATP) disodium salt hydrate, rhodamine B and quinacrine were obtained from Sigma-Aldrich (Gillingham, UK).

### Sample Preparation

Peptide solutions and W_2_R_2_ and W_3_R_3_ coacervates were prepared by mixing weighed amounts of peptide and water. In order to obtain W_2_R_2_ and W_3_R_3_ coacervates, the pH was fixed by titrating a solution containing 1.4 wt % NaOH.

Peptide:ATP coacervates were prepared by mixing weighted amounts of peptide and ATP and adding a weighted amount to water. The concentration of peptide and ATP was calculated in order to match the conditions of peptide:ATP charge 1:1 or 1:0.5.

A separate set of samples was prepared for confocal microscopy experiments. A solution containing 3 × 10^–4^ wt % rhodamine B (RhoB) was used as a solvent instead of water to stain the peptide. A solution containing 3 × 10^–3^ wt % quinacrine was used as a solvent, instead of water, to stain the ATP. Samples stained for simultaneous peptide and ATP staining were prepared by first dissolving the ATP in a solution containing 6 × 10^–3^ wt % quinacrine and then adding this solution to the peptide; the sample was completed by adding the necessary weight of 6 × 10^–4^ wt % RhoB solution.

### UV–Vis Absorption

The turbidity of the solutions was calculated from the UV–vis signal at 500 nm, a wavelength at which there is no intrinsic signal from the peptide, according to
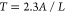
were *T* is the turbidity, *A* is absorbance at 500 nm and *L* is the optical path length in cm.

UV–vis spectra were measured using a Varian Cary 300 Bio UV–vis spectrometer with a 10 mm light path Quartz cell. The turbidity *T* was also measured by loading the samples in a 96-well plate and reading the absorbance using a Molecular Devices Spectramax 340PC plate reader UV–vis spectrometer.

### Electrophoretic Mobility (Zeta Potential)

The zeta potential was measured using a Zetasizer Nano ZS from Malvern Instruments. An aliquot 1 mL of sample was placed inside a disposable folded capillary cell. The sample was left to equilibrate for 120 s before measuring the zeta potential, using an applied voltage of 50.0 V. The results presented are the average over three measurements.

### Circular Dichroism (CD) Spectroscopy

Far-UV CD spectra were collected using a Chirascan spectropolarimeter (Applied Photophysics, Leatherhead, UK) equipped with a thermal controller. Spectra were recorded from 180 to 400 nm. Samples were mounted in a quartz cell with detachable windows, with 0.01 nm path length. CD signal from the samples was corrected by water background subtraction. Where necessary, the CD signal was smoothed using the Chirascan Software for data analysis. The residue of the calculation was chosen to oscillate around the average, to avoid artifacts in the smoothed curve. CD data, measured in mdeg, was normalized to molar ellipticity using the molar concentration of the sample and the cell path length.

### Optical Microscopy

For observation of coacervates, solutions were mounted in quartz cells with detachable windows, with 0.5 nm path length. Coacervate images were recorded using a GT Vision GXCAM camera. Precipitates, formed at the bottom of Eppendorfs in W_2_R_2_:ATP and W_3_R_3_:ATP solutions, were left to dry on a microscope slide. After drying, the hydrated sticky precipitates turned into solid samples. A flat surface was cut from the solid samples using a scalpel, and the texture of the surface was observed under the microscope within crossed polarizers. Images of solid precipitates were captured using a Canon G2 digital camera fitted to the microscope.

### Laser Scanning Confocal Microscopy (LSCM)

Imaging was performed using a Nikon A1 HD25/A1R HD25 confocal microscope. Solutions were prepared as detailed in the Sample Preparation section. Samples were loaded in a well of a μ-slide 8 well glass bottom plate. Experiments were performed using a Plan Apo λ 60 × oil lens or a Plan Apo λ 100 × oil lens. Pinhole sizes were 33.21, 97.06, or 255.43 μm. In solutions stained only with 3 × 10^–4^ wt % RhoB, the RhoB was excited at 561 nm and the emission was measured at 595 nm. In solutions stained only with 3 × 10^–3^ wt % quinacrine, the quinacrine was excited at 488 nm and the emission was measured at 525 nm. Finally, in samples stained with 3 × 10^–4^ wt % RhoB and 3 × 10^–3^ wt % quinacrine, the RhoB was excited at 561 nm and the emission was measured at 595 nm while the quinacrine was excited at 405 nm and the emission was measured at 450 nm. The shift in excitation and emission of quinacrine in the presence of RhoB might be associated with a FRET effect. A Nikon A1 HD25/A1R HD25 microscope was also used to record transmission detector (TD) images in a bright field transmission mode.

### Small-Angle X-Ray Scattering Experiments (SAXS)

SAXS experiments were performed on beamline B21^32^ at Diamond (Didcot, UK). The sample solutions were loaded into the 96-well plate of an EMBL BioSAXS robot and then injected via an automated sample exchanger into a quartz capillary (1.8 mm internal diameter) in the X-ray beam. The quartz capillary was enclosed in a vacuum chamber, to avoid parasitic scattering. After the sample was injected into the capillary and reached the X-ray beam, the flow was stopped during the SAXS data acquisition. To measure solid precipitates, samples were loaded on the multipurpose sample cell holder, specifically designed to measure highly viscous samples and solids at B21.^33^ Beamline B21 was operated with a fixed camera length (3.9m) and fixed energy (12.4 keV). The images were captured using a PILATUS 2 M detector. Data processing was performed using dedicated beamline software ScÅtter.

### Dynamic Light Scattering (DLS)

DLS experiments were done using an ALV/CGS-3 Compact Goniometer System with ALV/LSE-5003 correlator using vertical polarized incident light of wavelength 632.8 nm. Measurements were performed at an angle θ = 90° to the incident beam. The intensity autocorrelations functions were analyzed by the constrained regularized CONTIN method,^34^ to obtain distributions of hydrodynamic radius of the particle *R*_H_.

### Cryogenic-TEM (Cryo-TEM)

Imaging was carried out using a field emission cryo-electron microscope (JEOL JEM-3200FSC), operating at 200 kV. Images were taken in bright field mode and using zero loss energy filtering (omega type) with a slit width of 20 eV. Micrographs were recorded using a Gatan Ultrascan 4000 CCD camera. The specimen temperature was maintained at −187 °C during the imaging. Vitrified specimens were prepared using an automated FEI Vitrobot device using Quantifoil 3.5/1 holey carbon copper grids with a hole size of 3.5 μm. Just prior to use, grids were plasma cleaned using a Gatan Solarus 9500 plasma cleaner and then transferred into the environmental chamber of a FEI Vitrobot at room temperature and 100% humidity. Thereafter 3 μL of sample solution was applied on the grid and it was blotted twice for 5 s and then vitrified in a 1/1 mixture of liquid ethane and propane at temperature of −180 °C. The grids with vitrified sample solution were maintained at liquid nitrogen temperature and then cryo-transferred to the microscope.

### Charged Species Distributions

Distributions of charged species were calculated using the Henderson–Hasselbalch equation^35^ using the software HySS.^36^

### Quantum Mechanical Calculations

The renormalized charges for a Arg-Trp dimer were calculated using the R.E.D. server^37^ starting from a configuration with the two amino acid side chains manually aligned parallel. Geometry optimization and restrained electrostatic surface potential (RESP) charge calculations were performed using the R.E.D. server using Gaussian 16 at the HF/6-31G(d) level of theory. The RESP was converted to Amber force field parameters using antechamber, tleap within Amber and acpype.

### Molecular Dynamics Simulations

Molecular dynamics simulations were performed using Gromacs^38^ (versions 2023.2 or 2020.1-Ubuntu-2020.1-1). A total of 1600 molecules of W_2_R_2_ were randomly packed in a (24 nm)^3^ box using Packmol.^39^ The Gromacs input file was created using the Amber03 force field, the molecules were put in a larger cubic simulation box with sides 40.15 nm (with 1 nm between solute and box edge, corresponding to a W_2_R_2_ concentration 3 wt %) and the system was solvated with 2,101,430 TIP4P water molecules. The system was neutralized by adding 3200 Cl^–^ ions. The total number of atoms in the simulated system was 8,567,320. After energy minimization and 50 ps relaxation stages in the *NVT* and *NPT* ensembles, the final simulations were carried out in the *NPT* ensemble using a leapfrog integrator with steps of 2 fs up to 1540 ps. The temperature was maintained at 300 K using the velocity-rescale (modified Berendsen) thermostat^40^ with a coupling constant of 10 steps. The pressure was maintained at 1 bar using the Parinello–Rahman barostat^41^ and periodic boundary conditions were applied in all three dimensions. The Particle Mesh Ewald scheme^42,43^ was used for long-range electrostatics. Bonds were constrained using the LINCS algorithm^44^ and the Verlet cutoff scheme^45^ was used. Coulomb and van der Waals cutoffs were 1.0 nm. All arginine residues have a charge of +1 in the simulations.

## Results

Short peptide sequences containing tryptophan (W) and arginine (R) were designed to favor π–π and cation−π interactions potentially leading to liquid–liquid phase separation (LLPS), due to self-coacervation. We studied dipeptide WR, and blocky peptides tetrapeptide W_2_R_2_ and hexapeptide W_3_R_3_ (SI Figure S1). We examined potential LLPS using a combination of microscopy and scattering methods. Both W_2_R_2_ and W_3_R_3_ showed clear evidence for coacervation at sufficiently high pH. The peptides have calculated isoelectric points at pH 10.7, pH 12.1 and pH 12.4 for WR, W_2_R_2_, and W_3_R_3_ respectively. Titration curves were measured for W_2_R_2_ and W_3_R_3_ to determine p*K*_a_ values and the data are shown in SI Figure S2. The measured p*K*_a_ values were used to compute^36^ species distributions which are shown in SI Figure S3. This reveals that at high pH, where coacervation is observed, there are expected to be significant populations of species with charge −1, 0, and +1 (and +2 for W_3_R_3_). The presence of charged species is supported by electrophoretic mobility (zeta potential) measurements, to be discussed shortly. The population of neutral species facilities nonelectrostatic intermolecular interactions as discussed in the following.

**Figure 1:**
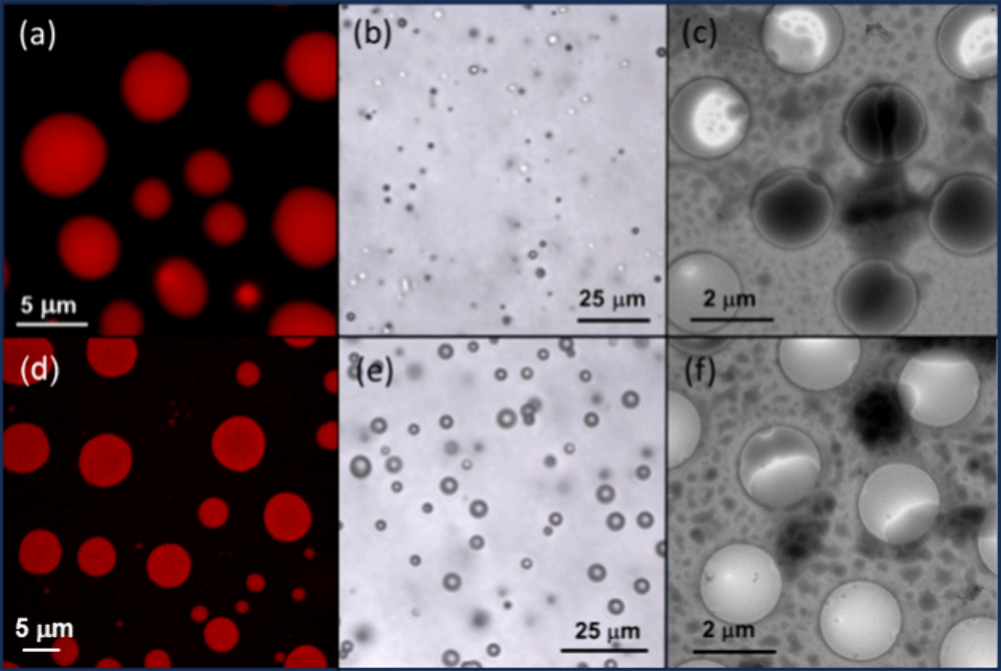
Data for 3 wt % W_2_R_2_ pH 12 droplets: (a) confocal and (b) optical microscopy and (c) cryo-TEM image. Data for 3 wt % W_3_R_3_ pH 12 droplets: (d) confocal and (e) optical microscopy and (f) cryo-TEM image. Samples for confocal microscopy were stained with 3 × 10^–4^ wt % RhoB. In the cryo-TEM images, droplets are irregular dark objects and the regular circular light regions are the TEM grid holes.

Representative confocal microscopy, optical microscopy and cryo-TEM images are shown in Figure 1. Additional data on the droplets is provided in SI Figures S4–S7. The coacervate droplets are polydisperse in size and there is a dynamic population. However, there is an upper bound to droplet size due to the associated interfacial tension, for which we introduce a model below based on surface charge effects. DLS was used to measure the hydrodynamic radius, *R*_H_, and the data in SI Figure S4 is consistent with coacervate droplet formation at high pH. The size distribution is narrower at higher concentration (3 wt %) and the data in SI Figure S4 also give an idea of the dynamic evolution of the droplet size distribution. SI Figures S5 and S6 show additional confocal microscopy images for W_2_R_2_ and W_3_R_3_ coacervates. The confocal and optical microscopy images in Figures 1a,b, and S5 show W_2_R_2_ coacervates with an average size ∼3500 nm. Cryo-TEM images (Figure 1c) for W_2_R_2_ reveal that larger coacervates coexist with smaller droplets with sizes ranging from ∼400 to ∼1700 nm. Similar features are observed for W_3_R_3_ (Figures 1d–f and S6). Sizes measured from microscopy techniques and DLS prove the high polydispersity of the coacervates for either sample, which ranges from hundreds to thousands of nm. The same result was systematically found for all the coacervates studied in this manuscript.

**Figure 2:**
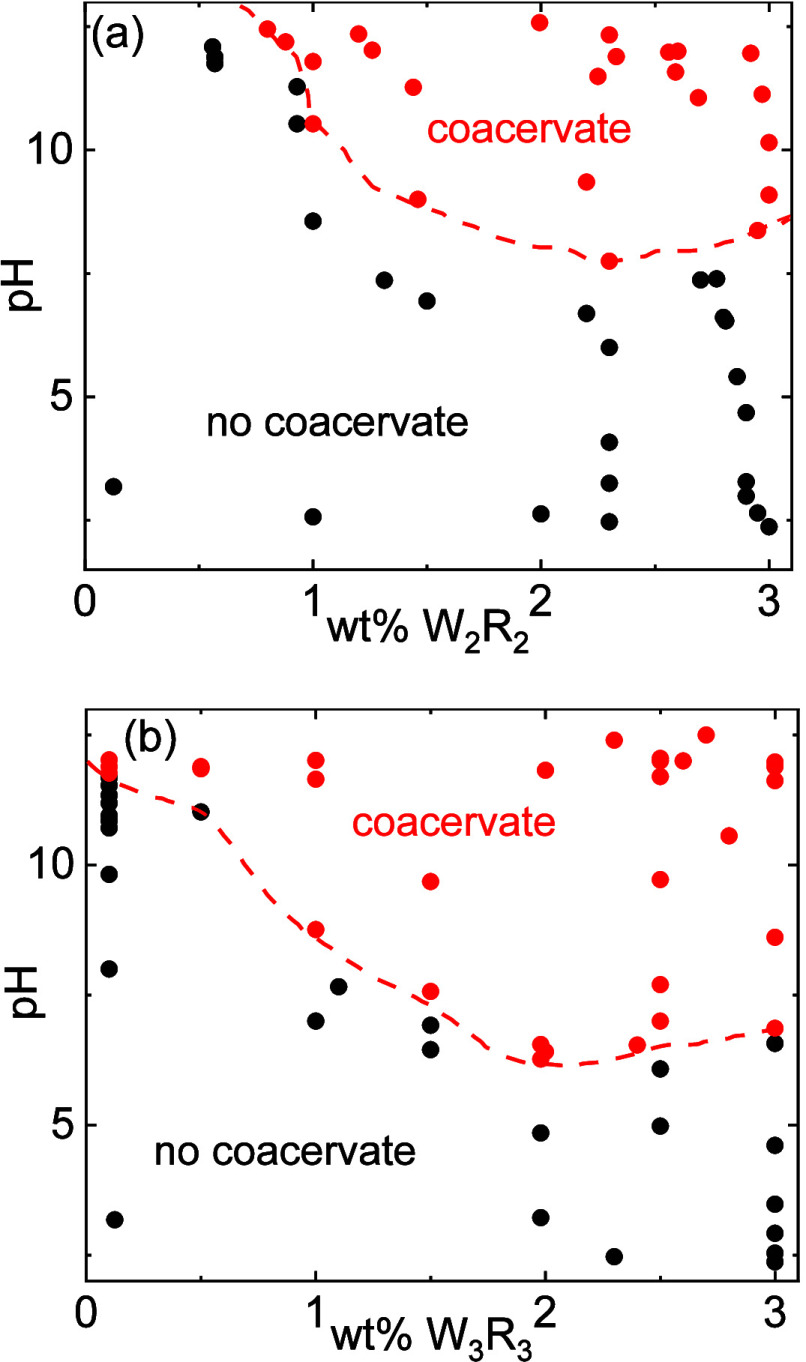
(a, b) Coacervate phase diagrams based on turbidity and optical microscopy measurements.

No evidence for LLPS due to self-coacervation was found for dipeptide WR. However, W_2_R_2_ and W_3_R_3_ show LLPS even in the absence of salt, in contrast to prior reports on longer peptide sequences containing W and R^26^ and proteins, where salt screens electrostatic interactions. Cation−π interactions between aromatic residues and arginine^6^ or hydrophobic interactions between arginine residues^25^ are then found to drive LLPS. For W_2_R_2_ and W_3_R_3_, phase diagrams showing conditions for coacervate (liquid droplet) formation were assembled via turbidity and optical microscopy measurements, as a function of peptide concentration and pH, and are presented in Figure 2. Images of turbid samples, as observed by the naked eye, together with representative turbidity data, from UV–vis experiments, are shown in SI Figure S7. At sufficiently high peptide concentration and high enough pH, the phase diagrams for W_2_R_2_ and W_3_R_3_ in Figure 2 show extensive areas of LLPS that leads to coacervate droplet formation and hence increased turbidity. The simple (self-)coacervation of these peptides is not driven by attractive electrostatic interactions, indeed measured zeta potentials under conditions where coacervates are formed are very low (SI Table S1), but is primarily driven by other intermolecular interactions (π–π stacking, cation−π stacking, as probed by circular dichroism (CD) spectroscopy and MD simulations to be discussed shortly). The small zeta potential values observed (SI Table S1) can be correlated to a small net charge on coacervate droplets according to a theoretical analysis, based on the Rayleigh analysis of the stability of charged droplets (to be discussed below).

**Figure 3:**
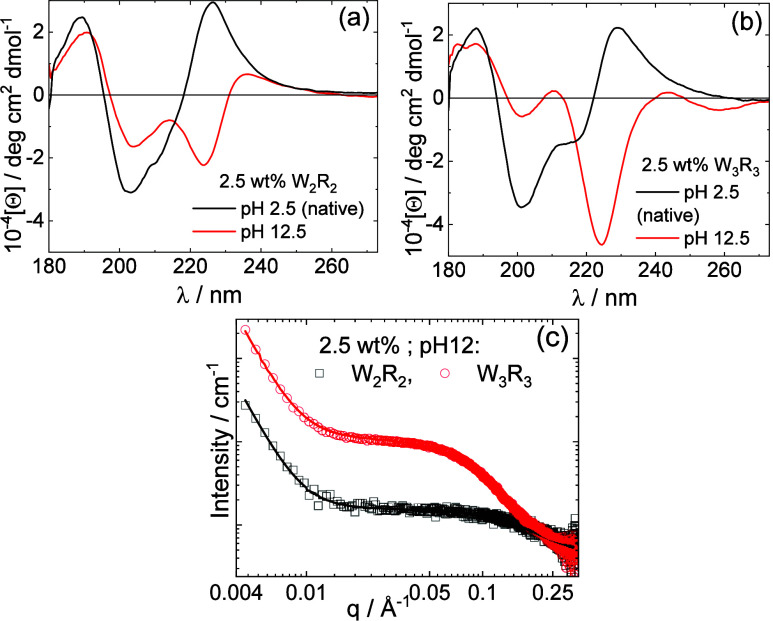
(a, b) CD spectra for solutions without coacervation (black lines, pH 2.5) and with coacervation (red lines, pH 12.5) and (c) SAXS data—open symbols: measured data (for ease of visualization only every 5th data point is shown) lines: form factor fits described in the text (fit parameters in SI Table S2).

CD spectra were measured to probe the molecular-level interactions involved in LLPS. In Figure 3, spectra obtained for native solutions (low pH; noncoacervate solutions; absence of LLPS) and at pH 12.5 (coacervate phase) are compared for W_2_R_2_ and W_3_R_3_. Spectra for noncoacervate samples at several concentrations, along with SAXS data showing that the peptides are present as monomers in solution are provided in SI Figure S8. Comparing spectra in Figure 3, coacervation clearly leads to large changes in the peptide conformation and chirality. In the absence of coacervation, the CD spectra show negative minima at 201–202 nm characteristic of a disordered conformation (along with a shoulder minimum near 214 nm for W_3_R_3_) and positive maxima at 226 nm (W_2_R_2_) or 229 nm (W_3_R_3_) which are due to the absorbance of the tryptophan residue.^46−49^ This peak is absent for coacervates and is replaced by a large negative peak at 223 nm. This indicates a change in the chiral environment of the tryptophan residues. This is associated with cation−π interactions, indeed the negative minimum at 223 nm has previously been assigned as a signal of such interactions for tryptophan–Cu^2+^ complexes.^50^ SAXS data was also obtained to probe nanostructure in solution.^51^ The data for coacervates are shown in Figure 3c and show a low wavenumber *q* power law intensity decay indicative of phase separation (LLPS) along with higher *q* scattering due to the monomer scattering. The data were fitted using a generalized Gaussian form factor model to describe the high *q* scattering (plateau and higher *q* decay to background) and a simple-power law *I*_s_*q*^–*n*^ (with *n* = 4, corresponding to Porod scattering from discrete objects,^51^ here droplets) for the low *q* scattering. The fit parameters are listed in SI Table S2 and show a radius of gyration *R*_g_ = 9.23 Å for W_2_R_2_ and significantly larger *R*_g_ = 18.95 Å for W_3_R_3_, while the value of Flory exponent ν for the Gaussian coil indicates a substantially compressed conformation, probably due to crowding.^52^ In contrast to the data for the coacervates, the SAXS profiles for samples at low pH do not show pronounced forward scattering indicative of LLPS. The data are shown in SI Figure 8d and can be described using generalized Gaussian form factors of monomers, with a generic structure factor term to account for the peaks observed for W_2_R_2_ and W_3_R_3_ near *q* = 0.1 A^–1^ which arise due to intermolecular correlations in the relatively high concentration conditions employed. The fit parameters are listed in SI Table 2.

**Figure 4:**
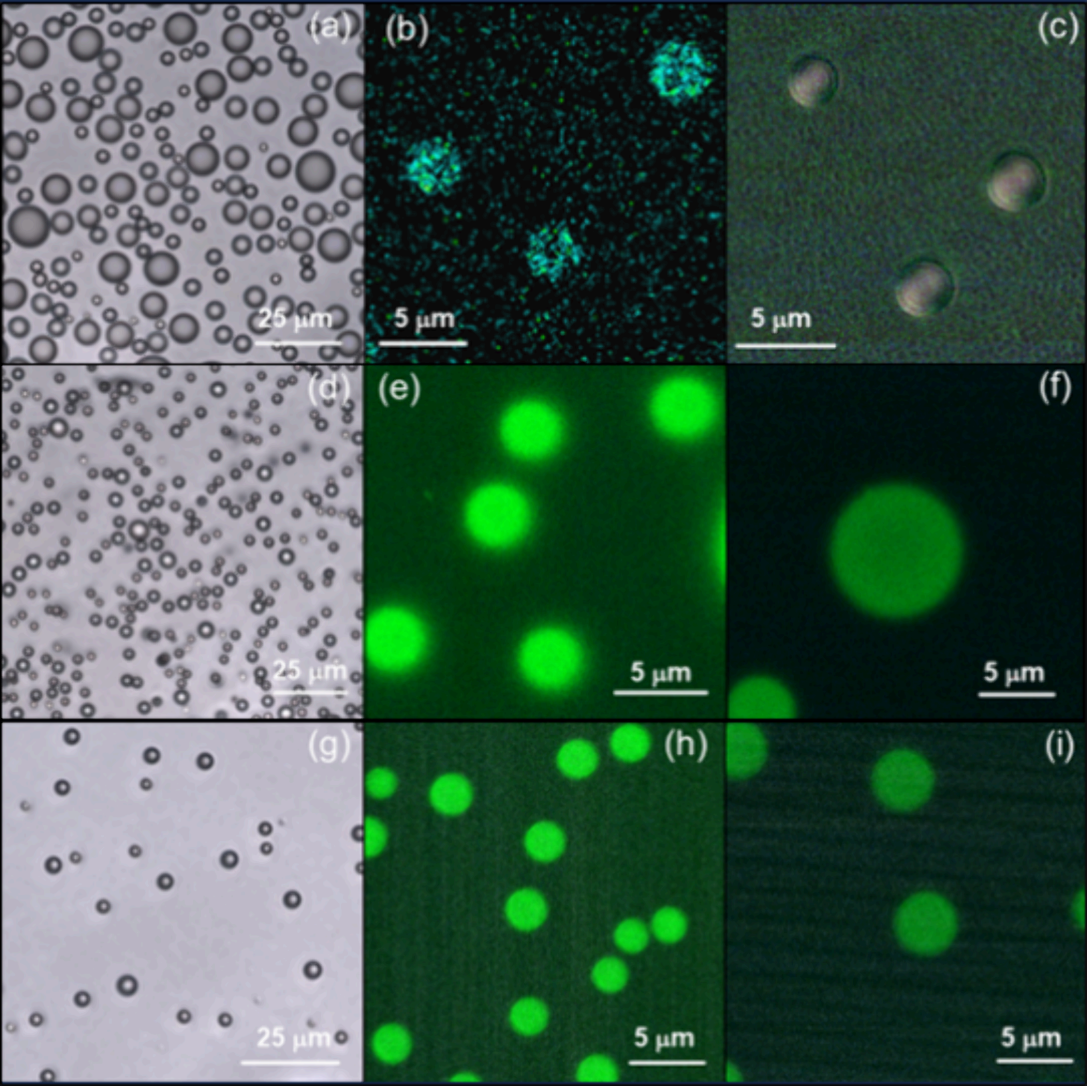
Microscopy images for samples containing peptide: ATP at charge ratio 1:1. (a) optical, (b) confocal, and (c) overlap of confocal and transmission images for 3 wt % WR:2.3 wt % ATP. (d) optical and (e, f) confocal microscopy images for 3 wt % W_2_R_2_:2.4 wt % ATP supernatant. (g) Optical and (h, i) confocal microscopy images for 3 wt % W_3_R_3_:2.4 wt % ATP supernatant. Samples for confocal microscopy were stained with 3 × 10^–3^ wt % quinacrine.

**Figure 5:**
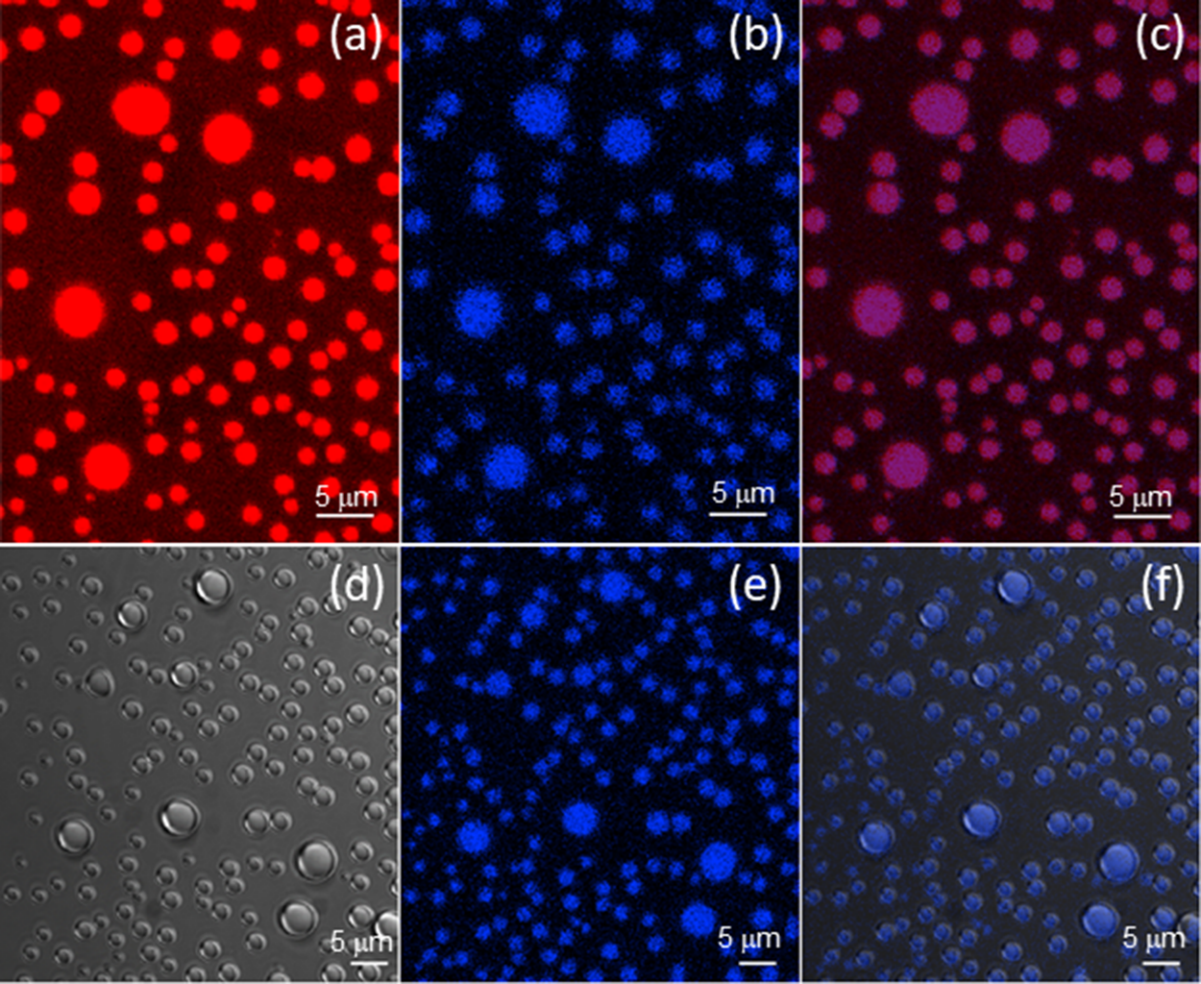
Confocal microscopy and transmission images for coacervates in supernatant of 3 wt % W_2_R_2_:2.4 wt % ATP charge ratio 1:1. Sample stained with 3 × 10^–4^ wt % RhoB and 3 × 10^–3^ wt % quinacrine. (a) Fluorescence of RhoB, (b) fluorescence of quinacrine, (c) overlap of images (a and b), (d) transmission image, (e) fluorescence of quinacrine, and (f) overlap of figures (d and e).

Inspired to create minimal protocell mimics that incorporate nucleobases as well as peptides, we investigated potential LLPS in mixtures of WR, W_2_R_2_ and W_3_R_3_ with the metabolic energy source mononucleotide adenosine triphosphate (ATP). Here electrostatic interaction between the anionic ATP (with double negative charge) and the cationic arginine residues is expected to drive intermolecular interactions underpinning LLPS. Mixtures were studied with 1:1 or 1:0.5 charge ratios and data is shown in Figures 4, 5 and S9–S18. Charge ratios were calculated considering charge −2 for ATP and charge +1, +2, or +3 for WR, W_2_R_2_, and W_3_R_3_ respectively (applicable at pH 5–6, Figure S3).

In contrast to the absence of self-coacervation of WR, LLPS was observed for WR with ATP (LLPS giving rise to coacervate droplets; Figures 4a–c, S10, S14, and S17a) whereas W_2_R_2_ and W_3_R_3_ formed precipitates (SI Figures S9 and S17d,e), although coacervate droplets were present in the supernatant (Figures 4d–i, S11–S13, S15, S16, and S17b,c). In other words, W_2_R_2_ or W_3_R_3_ with ATP undergo liquid–solid phase separation as well as liquid–liquid phase separation.

Figure 4 shows representative confocal microscopy images obtained for 1:1 peptide:ATP mixtures. Samples used for confocal microscopy in Figure 4 were stained with the ATP-binding dye quinacrine,^53,54^ to study the distribution of ATP within the droplets. Figure 4b,c show WR:ATP droplets arising from LLPS, while Figure 4e,f,h,i show coacervate droplets in the supernatant for mixtures containing W_2_R_2_ or W_3_R_3_ respectively. Confocal microscopy images in Figure 4 suggest that ATP is homogeneously distributed within the coacervates, and that W_2_R_2_:ATP or W_3_R_3_:ATP coacervate droplets are more compact than WR:ATP droplets.

For further confocal microscopy experiments, samples were simultaneously stained with RhoB and quinacrine, in order to observe the distribution of peptide and ATP within the coacervate droplets. Figure 5 shows confocal microscopy and transmission images for the supernatant of a 1:1 W_2_R_2_:ATP mixture, stained with RhoB and quinacrine. Figure 5a shows the fluorescence of the RhoB channel, while Figure 5b corresponds to the fluorescence of the quinacrine channel. The overlap of both images in Figure 5c proves the homogeneous distribution of peptide and ATP within the droplets. A similar conclusion can be attained from the overlap of the transmission image with the fluorescence of quinacrine (Figure 5d–f).

Additional confocal and optical microscopy images for peptide:ATP solutions, complementary to those displayed in Figure 4, are shown in SI Figures S10–S13. SI Figure S10 shows confocal (and transmission) microscopy images for 3 wt % WR:2.3 wt % ATP at a charge ratio 1:1, stained with RhoB and quinacrine, to study the distribution of WR and ATP in the sample. SI Figure S10c corresponds to the overlap of the quinacrine fluorescence image (SI Figure S10a) with that for RhoB channel (SI Figure S10b). The corresponding overlap of RhoB (SI Figure S10d) and transmission (SI Figure S10e) images is shown in SI Figure S10f. These results show a uniform distribution of peptide and ATP resulting from LLPS, consistent with Figure 4b,c. Additional confocal microscopy and transmission images for coacervates in the supernatant of W_2_R_2_ and W_3_R_3_ with ATP at a charge ratio 1:1 are shown in SI Figures S11 and S12. The image in SI Figure S11 is for a W_2_R_2_:ATP mixture stained with quinacrine; the 3D image reveals the shape of the droplets. For W_3_R_3_:ATP mixtures, the images in SI Figure S12, showing the fluorescence of quinacrine, suggest a preferential location of the ATP or dye at the walls of the droplets (SI Figure S12b). The images in SI Figure S13 are for a W_3_R_3_:ATP mixture stained with RhoB and quinacrine. The overlap of transmission and quinacrine fluorescence images (SI Figure S13a–c), together with the overlap of RhoB and quinacrine fluorescence images (SI Figure 13d–i) confirm that these have a homogeneous distribution of W_3_R_3_ and ATP.

Cryo-TEM images for droplets of coacervates in 1:1 mixtures of the three peptides with ATP are shown in SI Figures S14–S16. Cryo-TEM was used to check the size of small droplets. In fact, the cryo-TEM images in SI Figures S14–S16 show that LLPS produces a population of droplets a few nanometers in size as well as the larger droplets revealed by standard confocal microscopy and optical microscopy techniques.

SI Figure S17 shows optical microscopy images obtained for peptide:ATP mixtures at charge ratio 1:0.5. ATP mixtures containing WR were fully soluble, however solutions containing W_2_R_2_ or W_3_R_3_ separated into a supernatant and a precipitate. Precipitates, formed at the bottom of Eppendorf tubes in W_2_R_2_:ATP and W_3_R_3_:ATP solutions, were left to dry on a microscope slide. After drying, the hydrated sticky precipitates turned into solids. A flat surface was cut from the solid samples using a scalpel, and the texture of the surface was observed under the microscope within crossed polarizers. Representative polarized optical microscopy (POM) images for the precipitates for ATP mixtures with W_2_R_2_ at 1:0.5 charge ratio presented in Figures S17d,e reveal that some droplets have been “frozen” in the “matrix” of the dried precipitate. The POM images show birefringence with a texture around the droplet edge, and also droplets coexisting with fibrillar structures.

We again used CD spectroscopy to probe the effect of ATP on the conformation and chirality of the peptides (in supernatant solutions). The spectra are shown in SI Figure S18. The CD spectra for the peptides alone are presented in SI Figure S8. The spectrum for a 2.4 wt % ATP solution (SI Figure S18d) shows a negative minimum at 207 nm, a smaller negative minimum at 249 nm and a positive maximum at 267 nm. This spectrum is similar to that previously reported for ATP at low pH,^55,56^ conditions which lead to the aggregation of ATP, which is also enhanced in the presence of metal ions.^56,57^ In the presence of ATP, the spectra for the mixtures show similar shapes but greatly reduced molar ellipticity compared to those for the peptides themselves (SI Figure S8), particularly for the spectra for the lower ATP charge ratio 1:1 mixtures. The molar ellipticity is also greatly reduced compared to that for solutions of ATP at the same concentration and the peak positions differ. These observations suggest both that ATP binding leads to a considerable reduction in chiral ordering of the peptides, and that this binding leads to a loss of ATP chirality. Concerning the latter point, the positive maximum in the spectrum at 267–270 nm which is characteristic of ATP aggregation^57^ is lost in the mixtures (especially with W_2_R_2_ and W_3_R_3_). This suggests that ATP aggregates are disrupted by binding to peptides.

**Figure 6:**
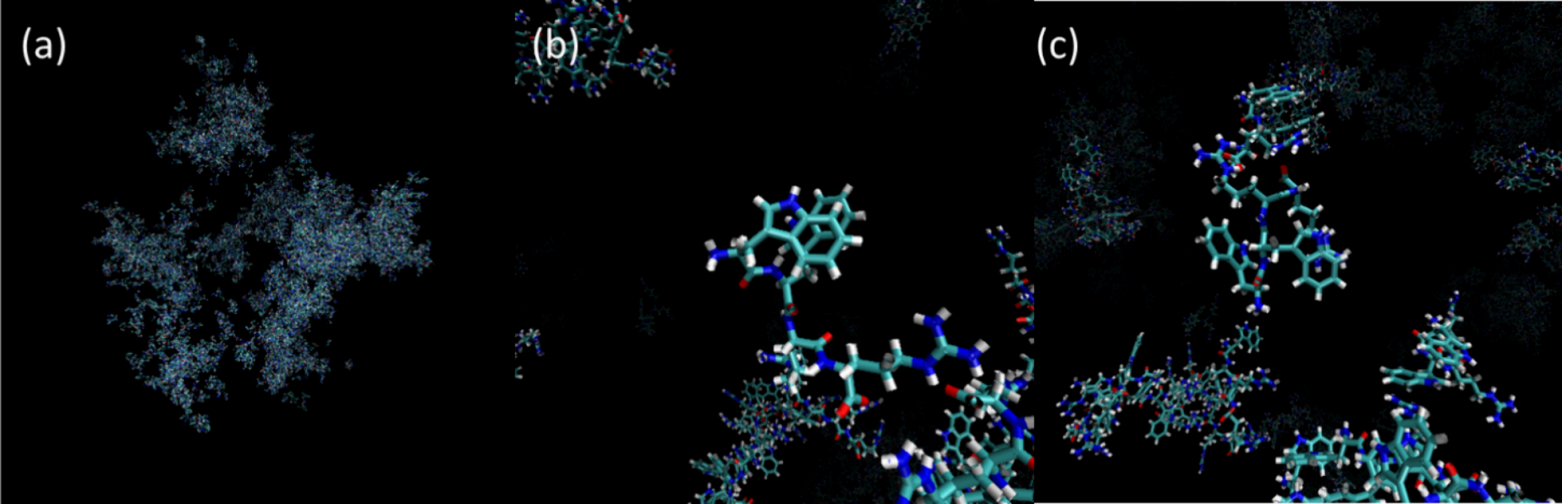
Images from MD simulation for W_2_R_2_: (a) snapshot showing aggregate domains after 1000 ps, (b) highlight showing (center) W–W π–π stacking, and (c) highlight showing (center) R–W cation−π stacking.

To gain insight into molecular interactions that may drive LLPS (coacervation), the self-aggregation of W_2_R_2_ was modeled through atomistic molecular dynamics (MD) simulations). Starting from a state comprising a random box of 1600 W_2_R_2_ molecules in water, the time-dependent formation of irregular aggregate structures with the appearance of phase-separated domains was observed (Figures 6, S19, and SI Movie S1) Convergence of the simulations is indicated through plots of the peptide radius of gyration (*R*_g_) and root-mean-square deviation (RMSD) of peptide atom positions, as shown in SI Figure S20. To probe the molecular interactions underpinning the aggregation process, radial distribution functions (RDFs) were analyzed (SI Figure S21), including those for arginine/tryptophan interactions shown in SI Figure S21b. This shows peaks due to W–W π–π stacking (*r* = 0.43 nm) Also present are peaks due to anion−π interactions and cation−π interactions (*r* = 0.62 and *r* = 0.70 nm respectively).^27,58^ A population of anions is expected due to charged peptide C termini as apparent in the species distributions in SI Figure S4. The interaction between the guanidinium group of arginine and the electron-rich π-system of tryptophan can renormalize the charge in the latter and this was calculated via quantum mechanical restrained electrostatic surface potential calculations (SI Table S3) as Amber force field parameters. The radial distribution functions shown in SI Figure S21c,d show the expected preferential location of water and Cl^–^ counterions molecules around Arg guanidinium atoms. The time-dependent development of aggregates is also indicated by cluster analysis in the MD simulations (SI Figure S22), clusters containing several hundreds of molecules are observed to form during the course of the MD simulation. These are clearly at a smaller scale than the experimentally observed coacervate aggregates, which are too large to model using atomistic simulations.

LLPS leads to the formation of droplets with an associated interfacial tension. The interfacial tension of biomolecular condensates has been determined from several methods including measurement of capillary fluctuations, wetting contact angles, optical trapping microrheology as well as droplet coalescence kinetics and viscosity.^59−64^ Here, we introduce a method to account for droplet interfacial tension based on surface charge arising from charged arginine residues. A charged droplet has zero charge inside (Gauss’ law), but the surface is charged. At the stability limit for a droplet of diameter *d* the surface charge is balanced by interfacial tension γ, and the net charge is given by^65−68^
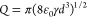
1Here ε_0_ = 8.85 × 10^–12^ F m^–1^ is vacuum permittivity. In terms of surface charge density, σ, the droplet diameter can be written as
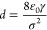
2

The surface charge density can be related to a measured zeta potential via the Grahame equation.^69,70^ For our system, with pH adjustment using sodium hydroxide and considering that peptides are supplied as TFA salts, the Grahame equation will take the form (at sufficiently low surface charge)^69,71^
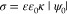
3Here ε is the relative permittivity (dielectric constant) κ is the inverse Debye length (the Debye length is here estimated to be 2.42 nm) and ψ_0_ is the surface potential. At pH 12 for 1 wt % W_3_R_3_, the zeta potential is measured as ψ_0_ = 1.1 mV (SI Table S1), this leads to an estimated surface charge σ = 0.31 mC m^–2^.

Using eq 2, it is thus possible to estimate the interfacial tension γ from the measured droplet size *d*. Taking *d* = 1.3 μm as an average value for W_3_R_3_ (cf. light scattering results in SI Figure S4b,d) and using the above value of σ leads to γ = 1.8 mN m^–1^. This may be compared to previous estimates for the interfacial tension of biomolecular condensates in the range 10^–4^–1 mN m^–1^.^64,72,73^ These measurements for a variety of systems have been obtained using a range of invasive techniques including capillary adhesion,^73^ contact probe-AFM^72^ and micropipette aspiration,^74^ as well as noninvasive methods such as droplet shape analysis^59^ and optical trapping microrheology.^60^

The large dispersity in droplet size here (associated with a kinetic phase separation process) implies a considerable variability in γ (eq 1), however the method introduced here is based on a simple noninvasive measurement (requiring only droplet size and zeta potential measurements). Also, the literature values quoted apply to droplets formed by phase-separating proteins, polyelectrolytes or cell nucleoli and we are not aware of prior estimations of interfacial tension for short peptide systems undergoing coacervation.

## Conclusions

In summary, the tetrapeptide W_2_R_2_ and hexapeptide W_3_R_3_ undergo self-coacervation under conditions of sufficiently high pH and concentration. These are among the shortest peptides yet shown to exhibit this type of phase behavior. CD spectroscopy indicates that cation−π interactions are characteristic of the coacervate state. The MD simulations confirm that the self-coacervation of W_2_R_2_ or W_3_R_3_ is driven by a combination of intermolecular interactions that includes π–π stacking of tryptophan and anion−π and arginine–tryptophan cation−π interactions. The latter are considered the strongest driving force for LLPS in low complexity domain proteins.^75^ Coacervate droplets are charge stabilized and we show that it is possible to estimate the interfacial tension from zeta potential measurements. We also showed that the dipeptide WR does not form simple coacervates under the conditions examined. However, complex coacervates were observed due to electrostatic complexation with ATP.

We have developed minimal simple coacervate peptides that undergo LLPS in the absence of added salts or crowding agents. These are expected to be valuable for further development as model systems for synthetic biology, biomedicine and other applications. In addition, we present an electrostatically stabilized complex coacervate system of dipeptide WR with mononucleotide ATP that could be a useful basis for future research on protocell models^11^ that contain truly minimal peptide and nucleotide components.

We present a method to obtain interfacial tension of coacervate droplets from DLS (hydrodynamic radius) and zeta potential measurements. Our work may also stimulate developments in theoretical modeling of LLPS. Approaches such as the random phase approximation (RPA)^76,77^ are not suitable to model small blocky peptides such as WR, W_2_R_2_, and W_3_R_3_. Field theoretic approaches have been employed for blocky oligomers (with 50 residues, much longer than our peptides)^77^ although to date only electrostatic interactions have been considered. It would be interesting to extend this approach to allow for π–π stacking and cation−π interactions. Future experimental work could include investigations of active coacervate formation induced by enzymatic cascades, e.g., from (enzymatic) coupling of individual amino acids^78^ in the presence of ATP activated enzymatically as a fuel for protocell growth.^79^ Related work could investigate whether protocells could be prepared thermochemically via coacervation, starting from a prebiotic soup of certain amino acids and mononucleotides in the absence of enzymes.

## Supporting Information Available

Molecular structures of peptides, titration curves and calculated species distributions, data for peptide coacervates from light scattering, additional confocal microscopy, cryo-TEM, and turbidity measurements; CD and SAXS data for peptides in noncoacervate state; confocal microscopy, cryo-TEM, and SAXS data for ATP:peptide mixtures; data from MD simulations for W_2_R_2_: analysis of RMSD and *R*_g_ to confirm equilibration, cluster size distributions; and QM calculation of renormalized tryptophan charges, SAXS data fitting parameters, and zeta potential measurement data (PDF)
Molecular dynamics simulation showing phase separation in 3 wt % W2R2 solution (0–1000 ps) (MPG)

## Supplementary Material

bm4c00738_si_001.pdf

bm4c00738_si_002.mpg
