# Supplementary material for: Minimal Peptide Sequences That Undergo Liquid–Liquid Phase Separation via Self-Coacervation or Complex Coacervation with ATP
Source: Biomacromolecules. 2024 Jul 27;25(8):5321–31. doi: 10.1021/acs.biomac.4c00738 (PMC11323023; doi:10.1021/acs.biomac.4c00738)
Supplement: Supplementary file 1 — bm4c00738_si_001.pdf [file bm4c00738_si_001.pdf]

Supporting Information

Minimal Peptide Sequences that Undergo Liquid-Liquid Phase Separation via Self- Coacervation or Complex Coacervation with ATP

Valeria Castelletto,[1] Jani Seitsonen,[2] Alice Pollitt[3], Ian W Hamley[1,*]

[1] *School of Chemistry, Food Biosciences and Pharmacy, University of Reading, Whiteknights, Reading RG6 6AD, U.K.*

[2] *Nanomicroscopy Center, Aalto University, Puumiehenkuja 2, FIN-02150 Espoo, Finland*

[3] *Institute for Cardiovascular and Metabolic Research, School of Biological Sciences, University of Reading, Reading RG6 6AS, U.K.*

* Author for correspondence. I.W.Hamley@reading.ac.uk

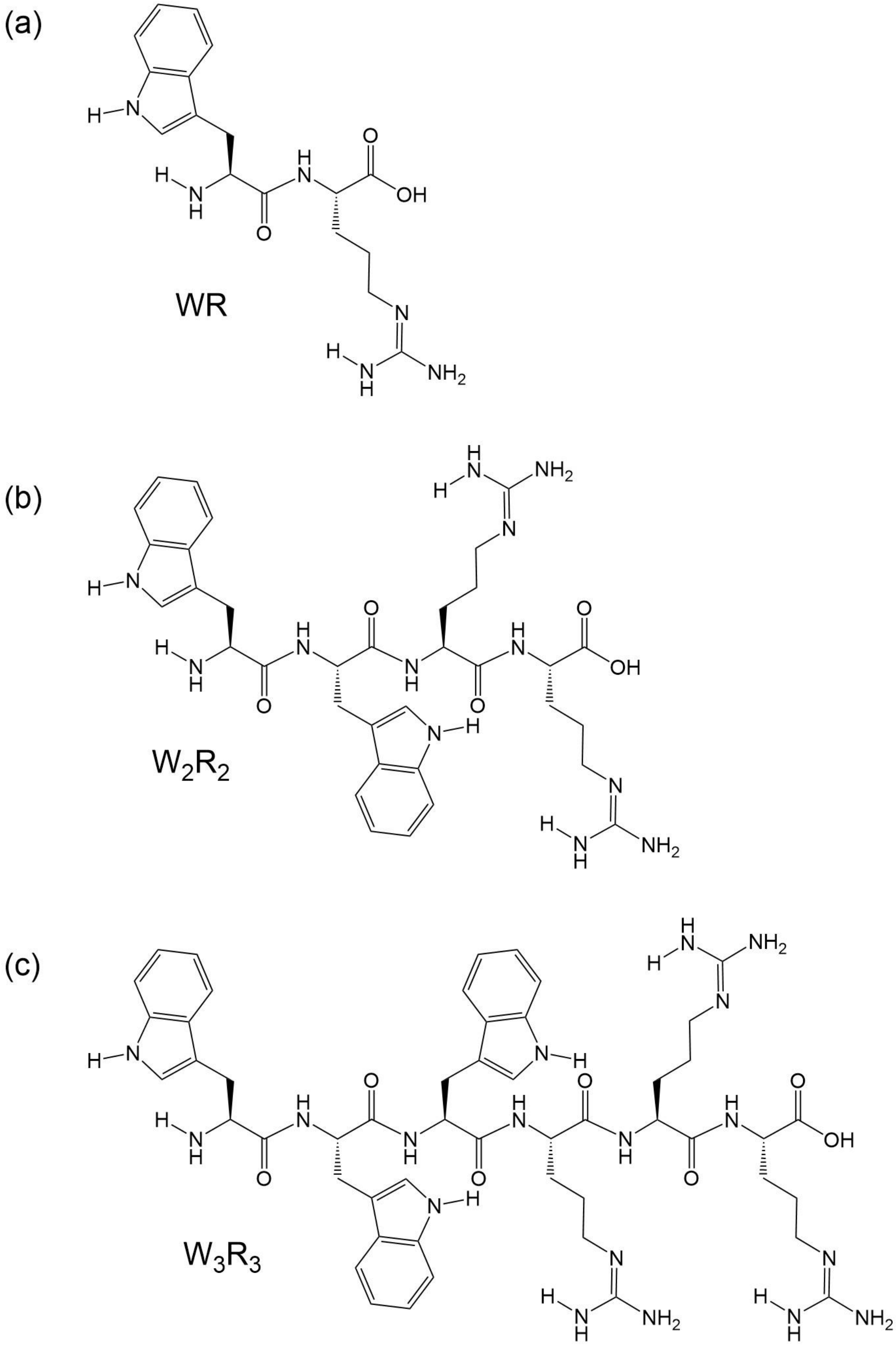

Fig.S1. Molecular structures, (a) WR, (b) W_2R_2, (c) W_3R_3.

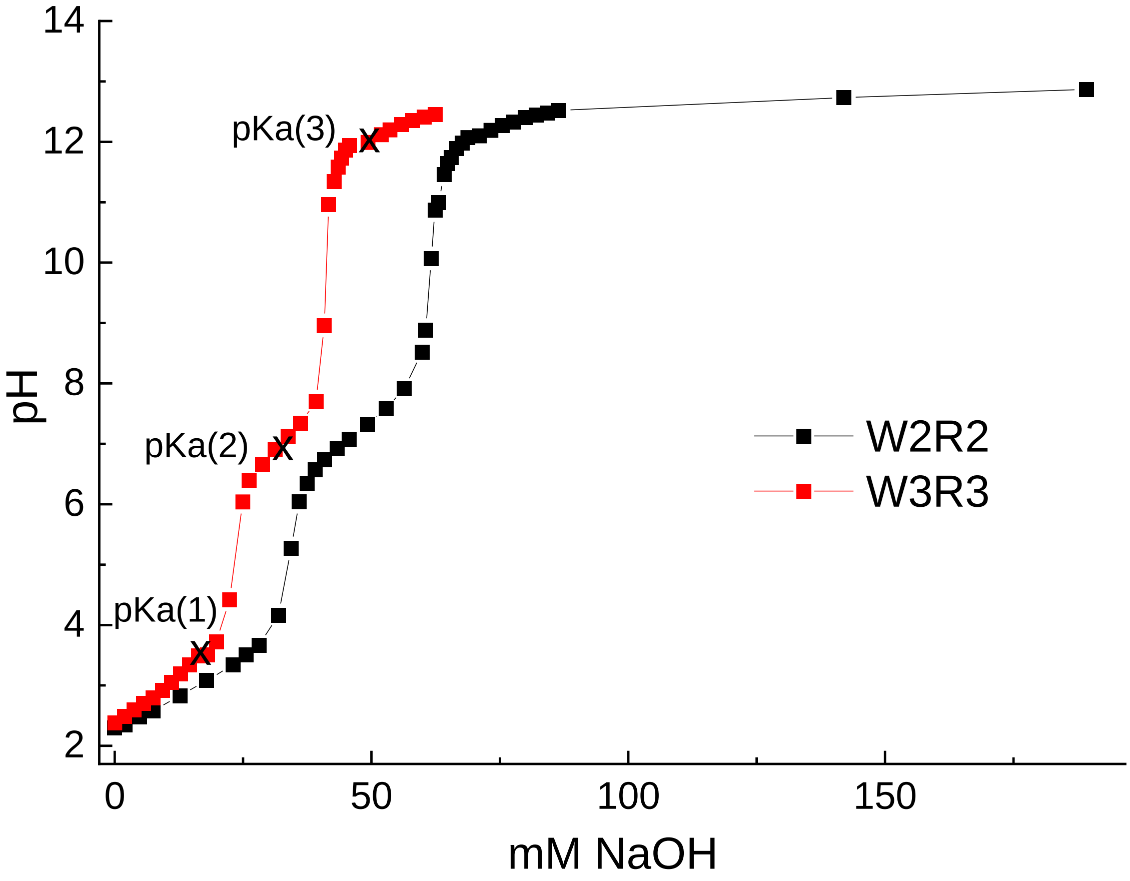

Fig.S2. Titration curves for 3 wt% solutions of W_2R_2 and W_3R_3 with indicated pKa values (the same within uncertainty for W_2R_2 as W_3R_3). Here pKa(1) = 3.5, pKa(2) = 7, pKa(3) = 12.

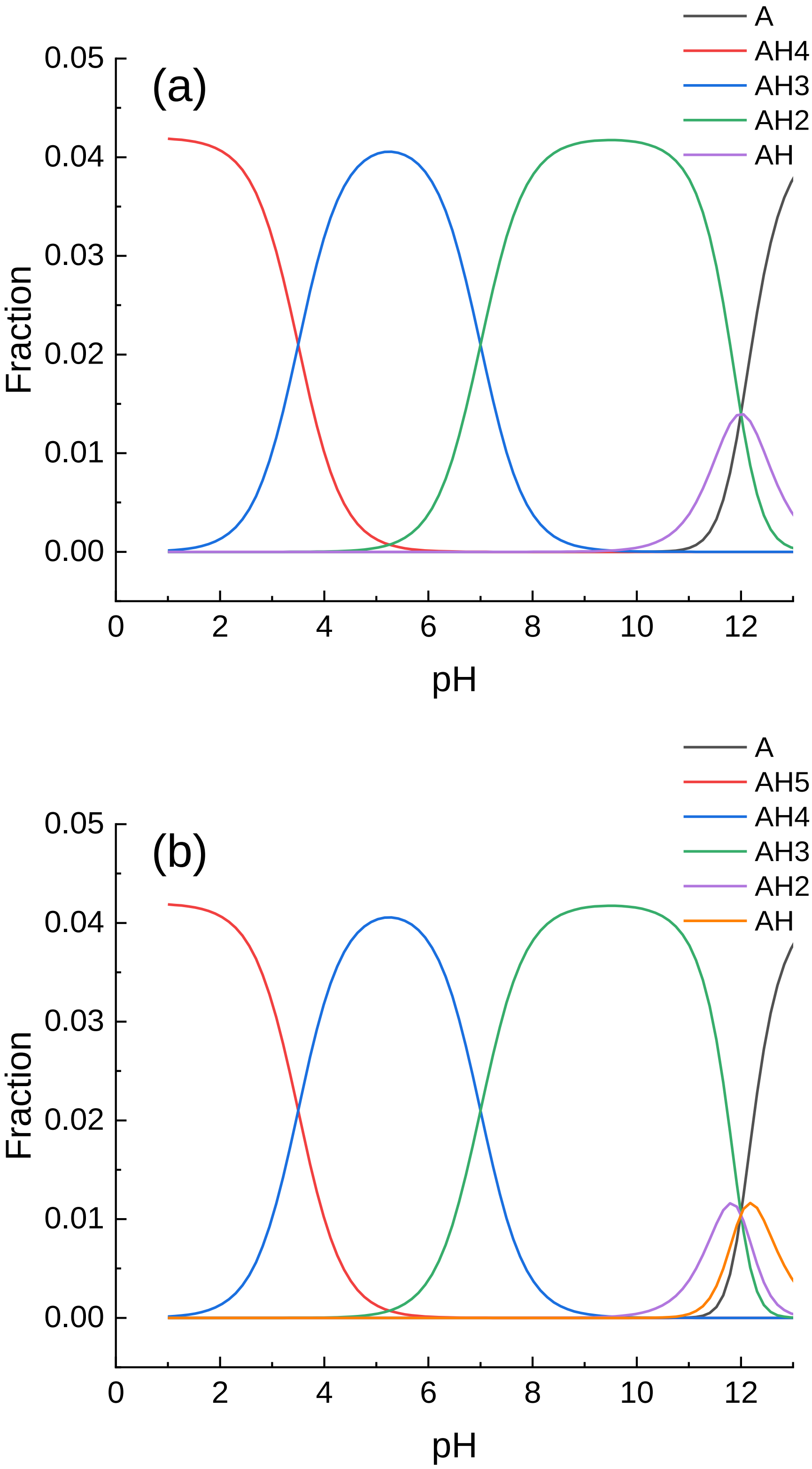

Fig.S3. calculated species distributions for 3 wt% solutions. (a) W_2R_2, (b) W_3R_3. Notation for W_2R_2: A: NH_2-W_2R_2-COO^-, AH: NH_2-$W_2R_2^+$-COO^-, AH2: NH_2-$W_2R_2^{++}$-COO^-, AH3: NH_3^+-$W_2R_2^{++}$-$COO^{-,}$ AH4: NH_3^+-$W_2R_2^{++}$-COOH. Notation for W_3R_3: A: NH_2-W_2R_2-COO^-, AH: NH_2-$W_3R_3^+$-COO^-, AH2: NH_2-$W_3R_3^{++}$-COO^-, AH3: NH_2-$W_3R_3^{+++}$-$COO^{-,}$ AH4: NH_3^+-$W_3R_3^{+++}$-COO^-, AH5: NH_3^+-$W_3R_3^{+++}$-COOH.

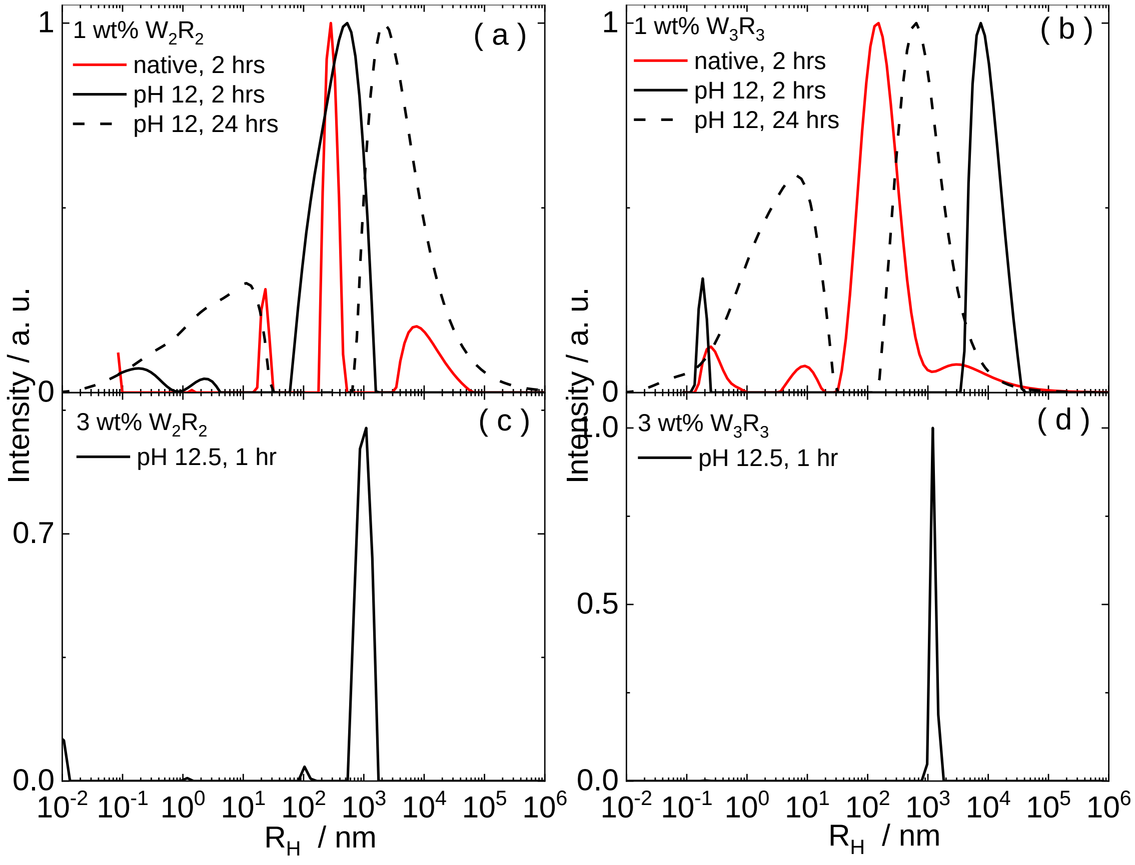

Fig. S4. Distribution of hydrodynamic radius R_H for coacervates or native solutions: (a) 1 and (c) 3 wt% W_2R_2; (b) 1 and (d) 3 wt% W_3R_3. The age of the sample and the pH are indicated in the graphs.

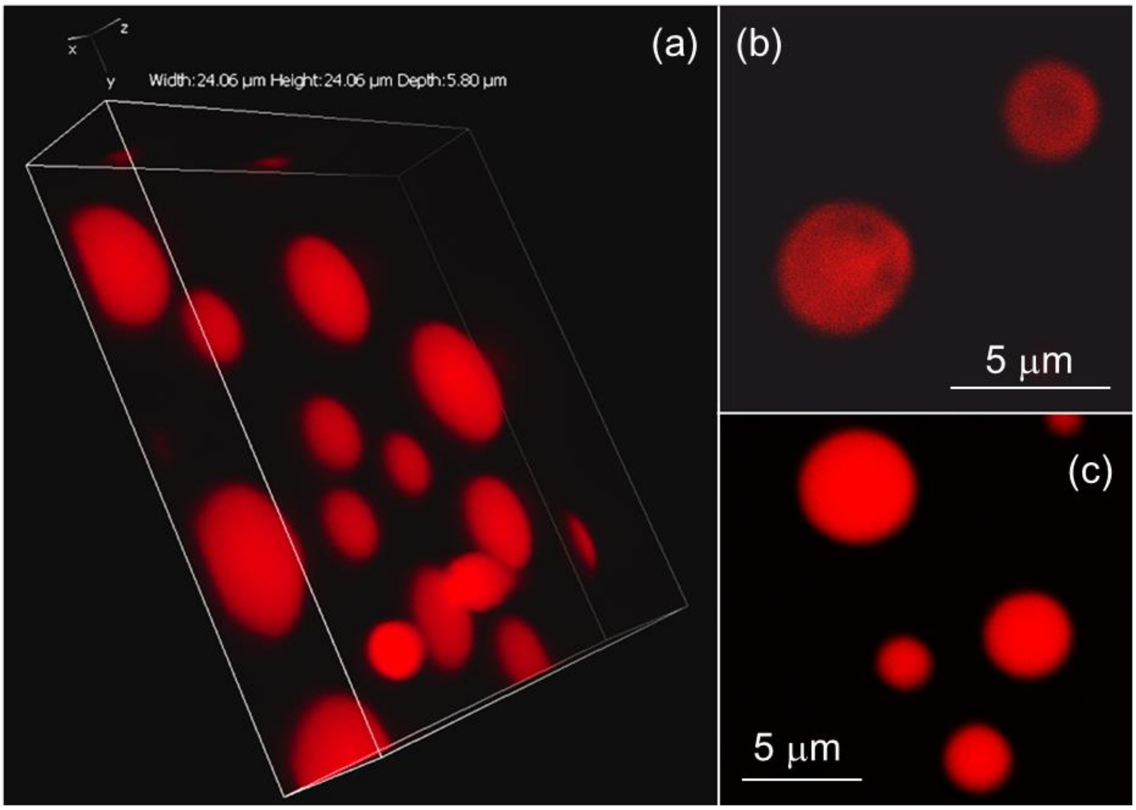

Fig. S5. (a-c) Confocal microscopy images for 3 wt% W_2R_2 at pH 12 (stained with 3 $\times 10^{-4}$ wt% RhoB).

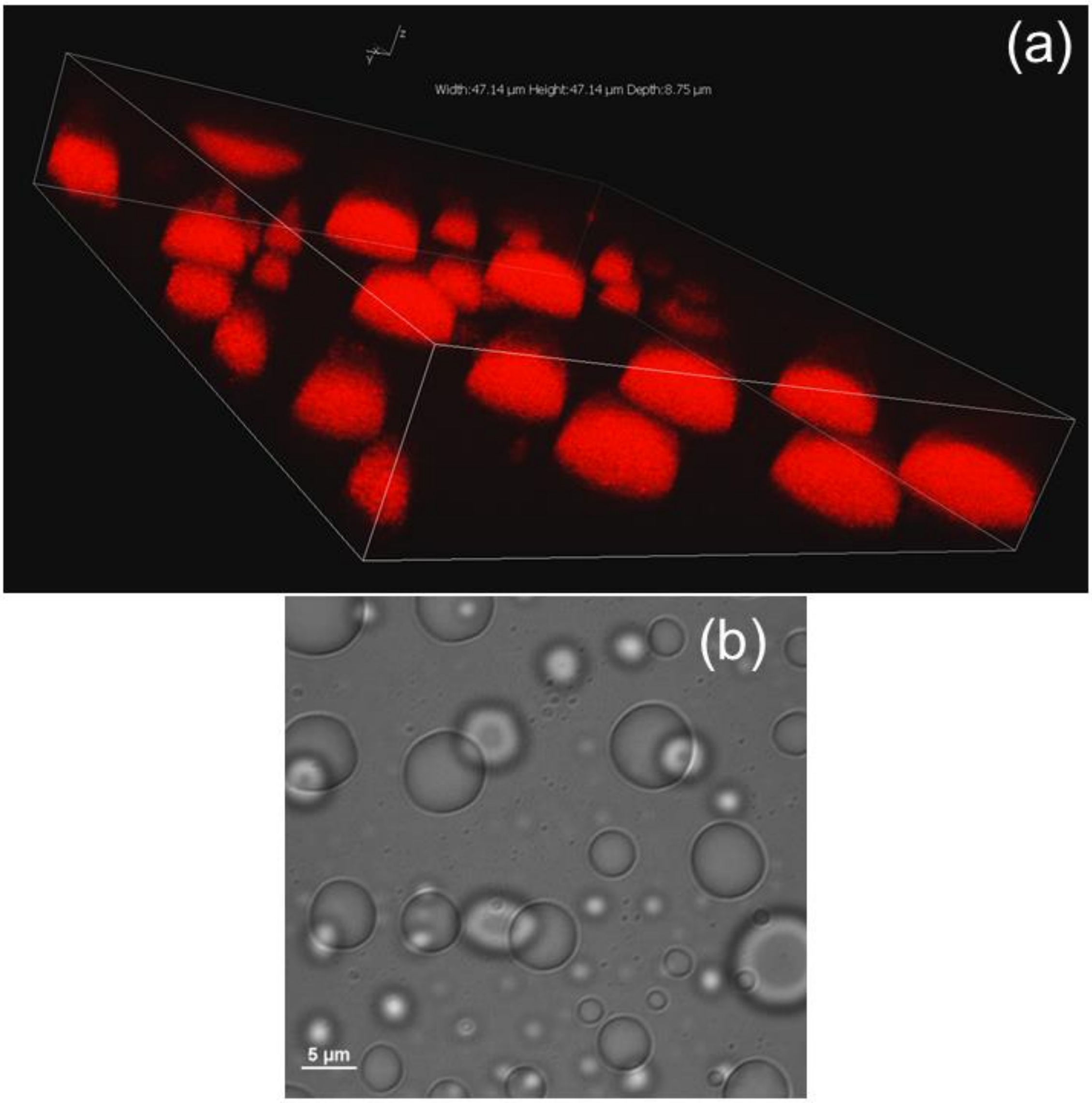

Fig. S6. (a) Confocal microscopy and (b) transmission detector images for 3 wt% W_3R_3 at pH 12 (sample stained with 3×10^{-4} wt% RhoB).

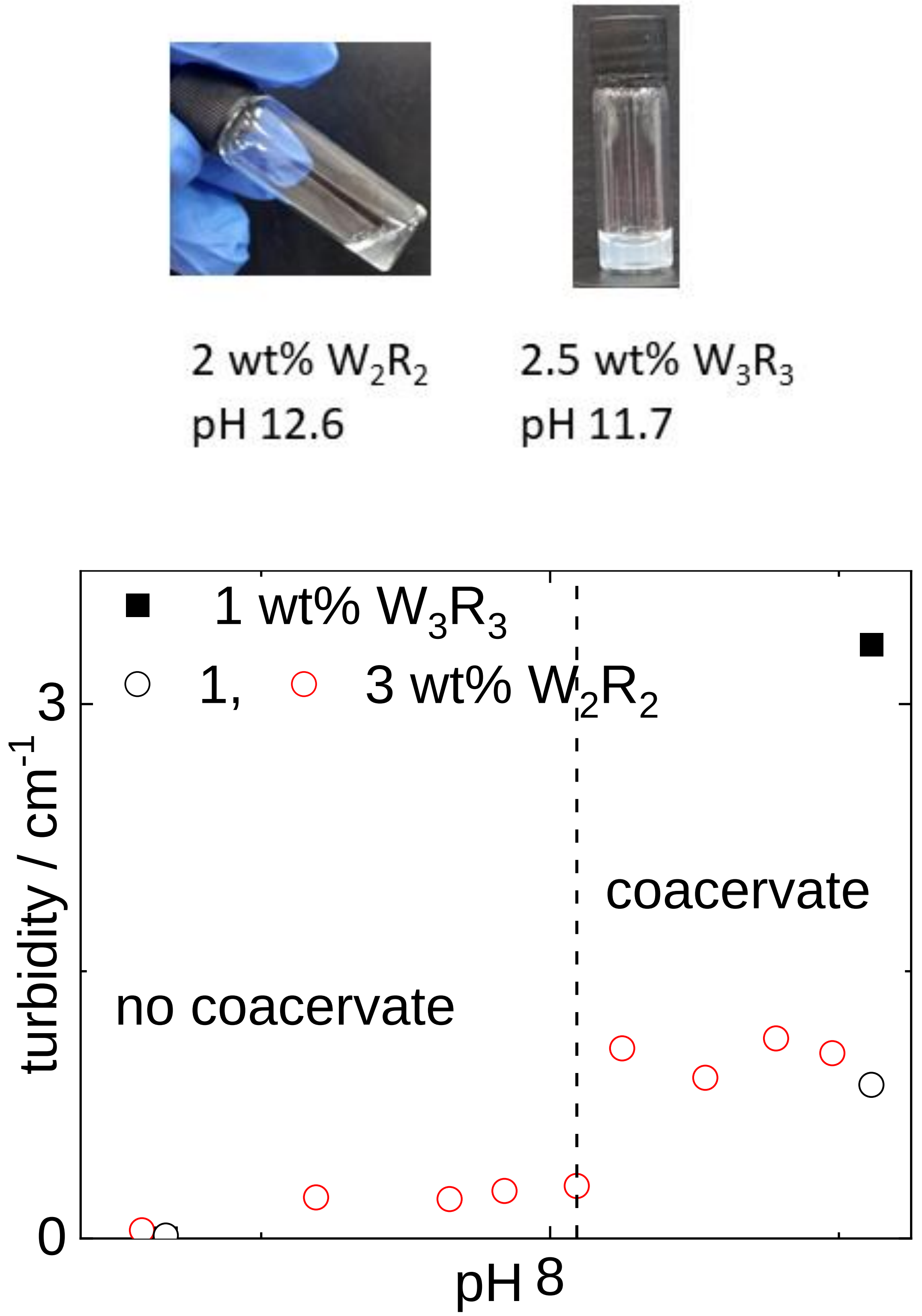

Fig. S7. Appearance of coacervate samples and representative turbidity values measured for W_2R_2 and W_3R_3 solutions as a function of the pH.

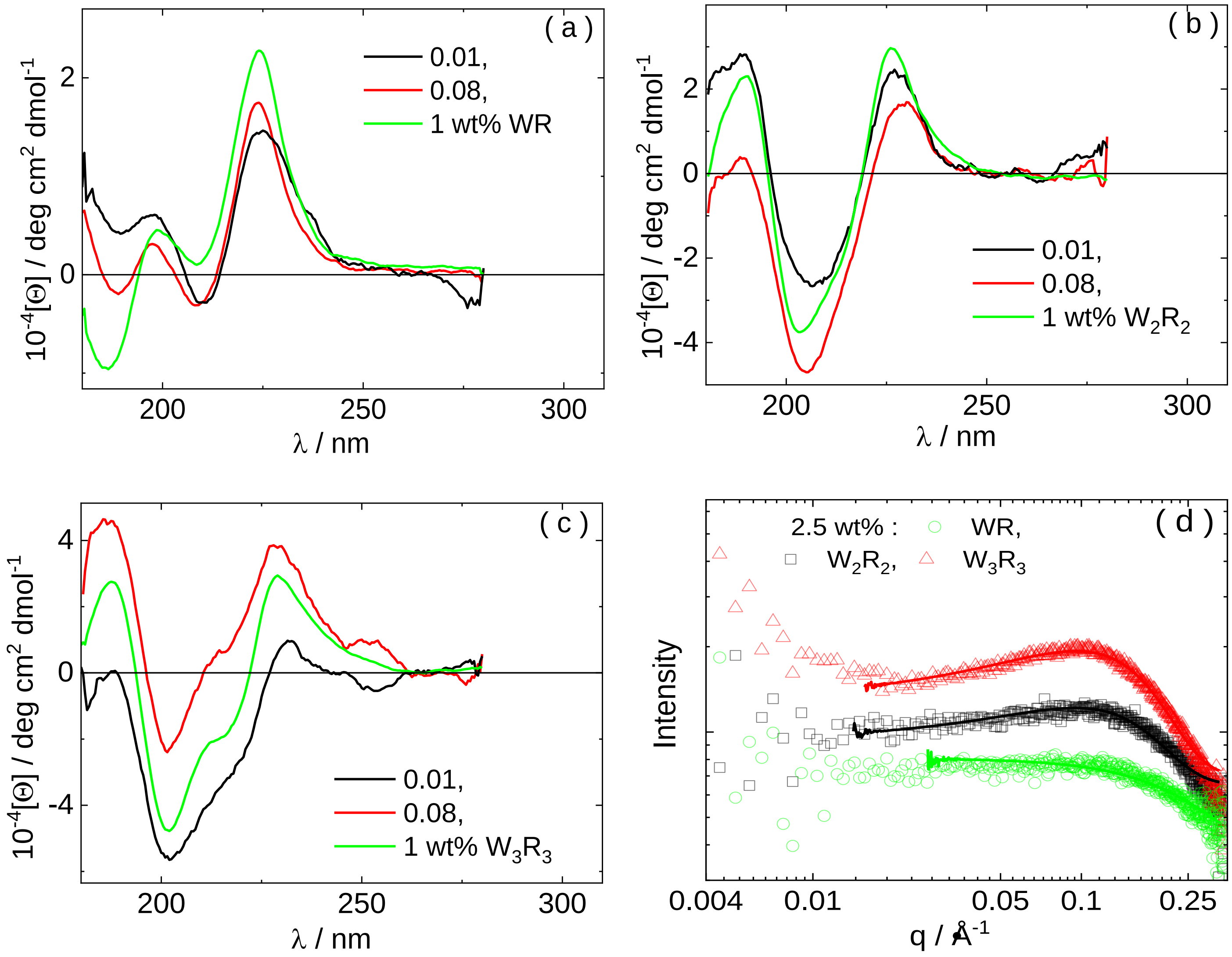

Fig.S8. (a-c) CD spectra for samples at native pH (pH 2.5) in the absence of coacervation and (d) SAXS data under the same conditions. Open symbols: measured data, lines: fits as described in the data (parameters in SI Table S2). For ease of visualization only every 5th data point is plotted.

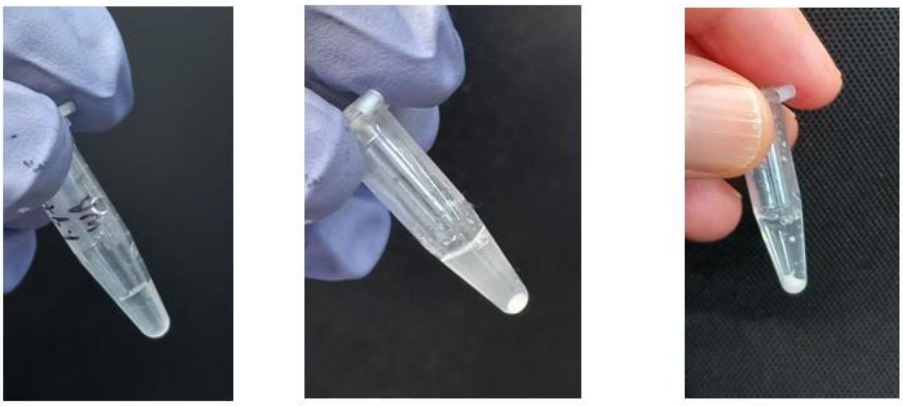

3 wt% WR: 2.3 wt% ATP in water. Charge ratio 1:1 WR:ATP

3 wt% W2R2: 2.4 wt% ATP in water. Charge ratio 1:1 W2R2:ATP

3 wt% W3R3: 2.4 wt% ATP in water. Charge ratio 1:1 W3R3:ATP

Fig.S9. Images of samples of three peptides in water in the presence of ATP (1: 1 charge ratio). (a) 3 wt% WR: 2.3 wt% ATP (b) 3 wt% W_2R_2: 2.4 wt% ATP, (c) 3 wt% W_3R_3: 2.4 wt% ATP.

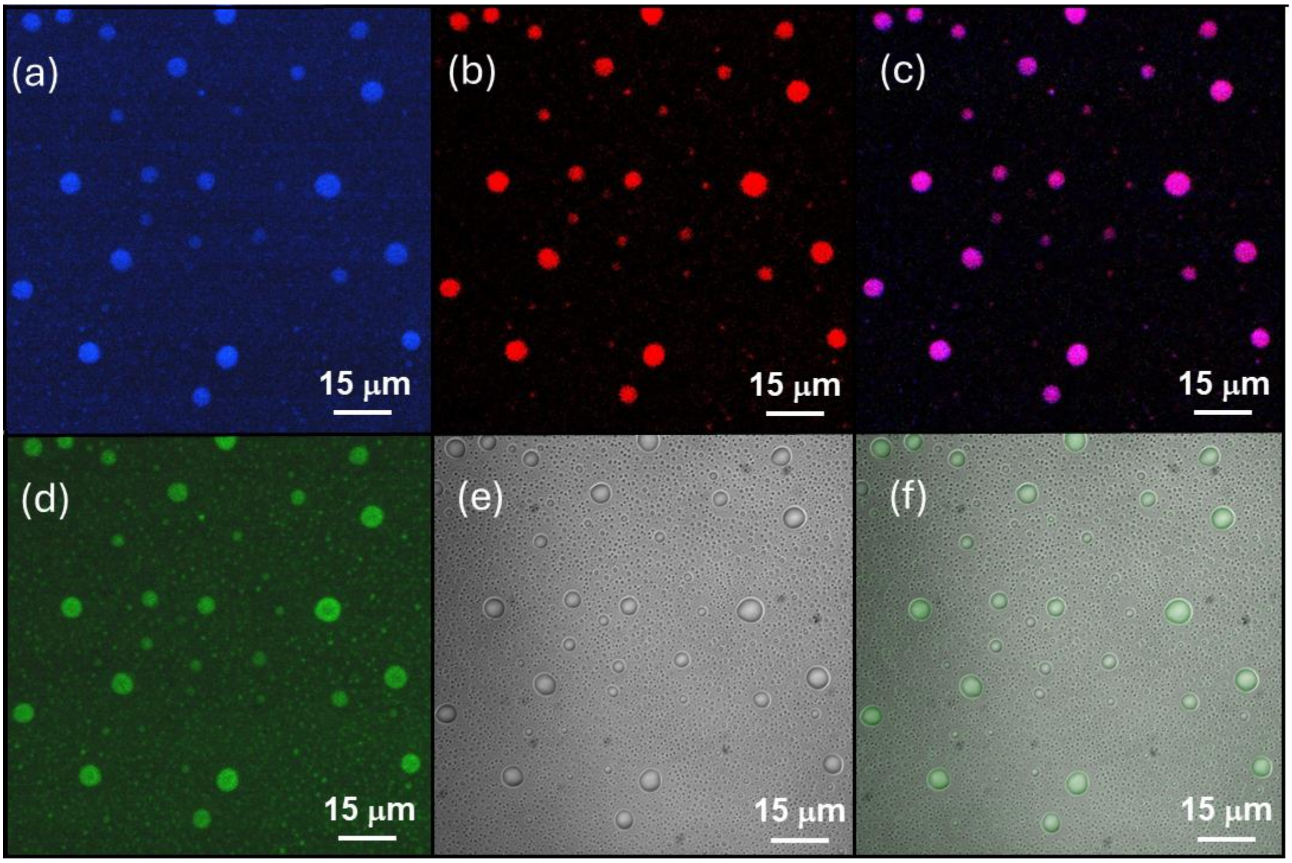

Fig S10. Confocal microscopy images for coacervates for 3 wt% WR:2.3wt% ATP charge ratio 1:1. The sample was stained with 3×10^{-4} wt% RhoB and 3×10^{-3} wt% quinacrine. Fluorescence of (a) quinacrine, (b) RhoB and (c) overlap of images (a) and (b). (d) Fluorescence of RhoB, (e) transmission image and (f) overlap of images (d) and (e).

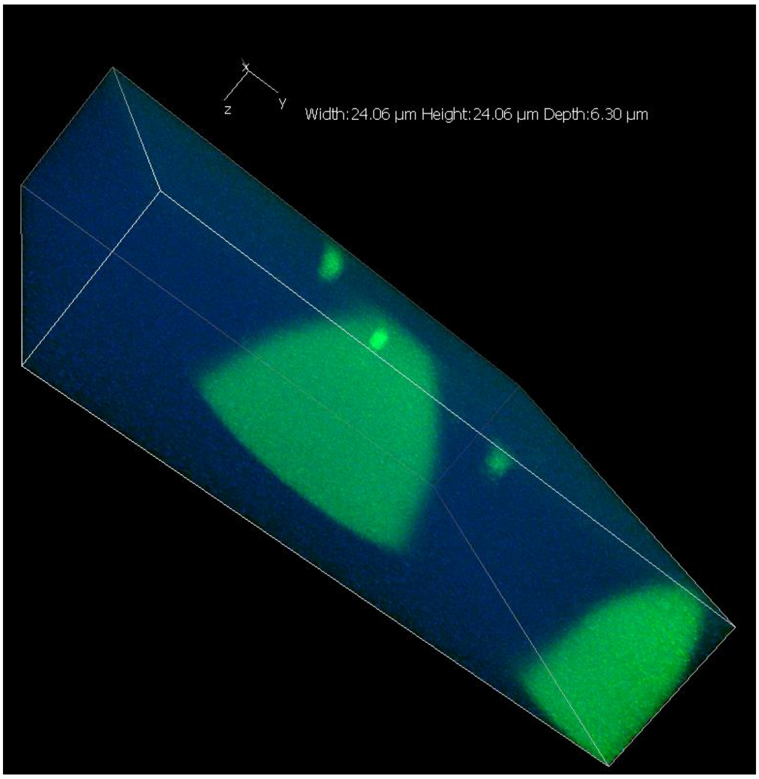

Fig.S11. Confocal microscopy image of coacervate droplets observed in the supernatant of 3 wt% W_2R_2:2.4wt% ATP charge ratio 1:1. Sample stained with 3×10^{-3} wt% quinacrine.

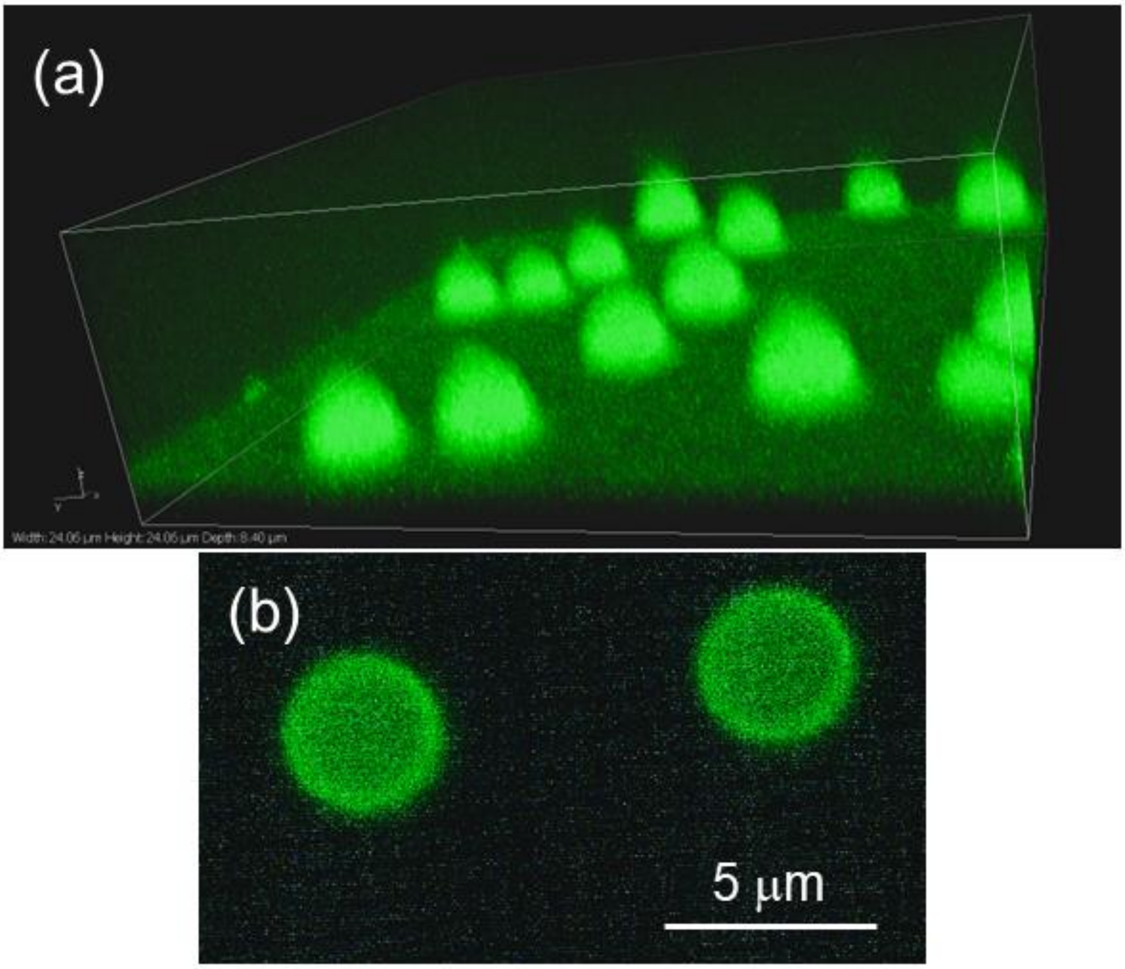

Fig.S12. (a-b) Confocal microscopy images of coacervate droplets observed in the supernatant of 3 wt% W_3R_3:2.4wt% ATP charge ratio 1:1. Samples stained with 3×10^{-3} wt% quinacrine.

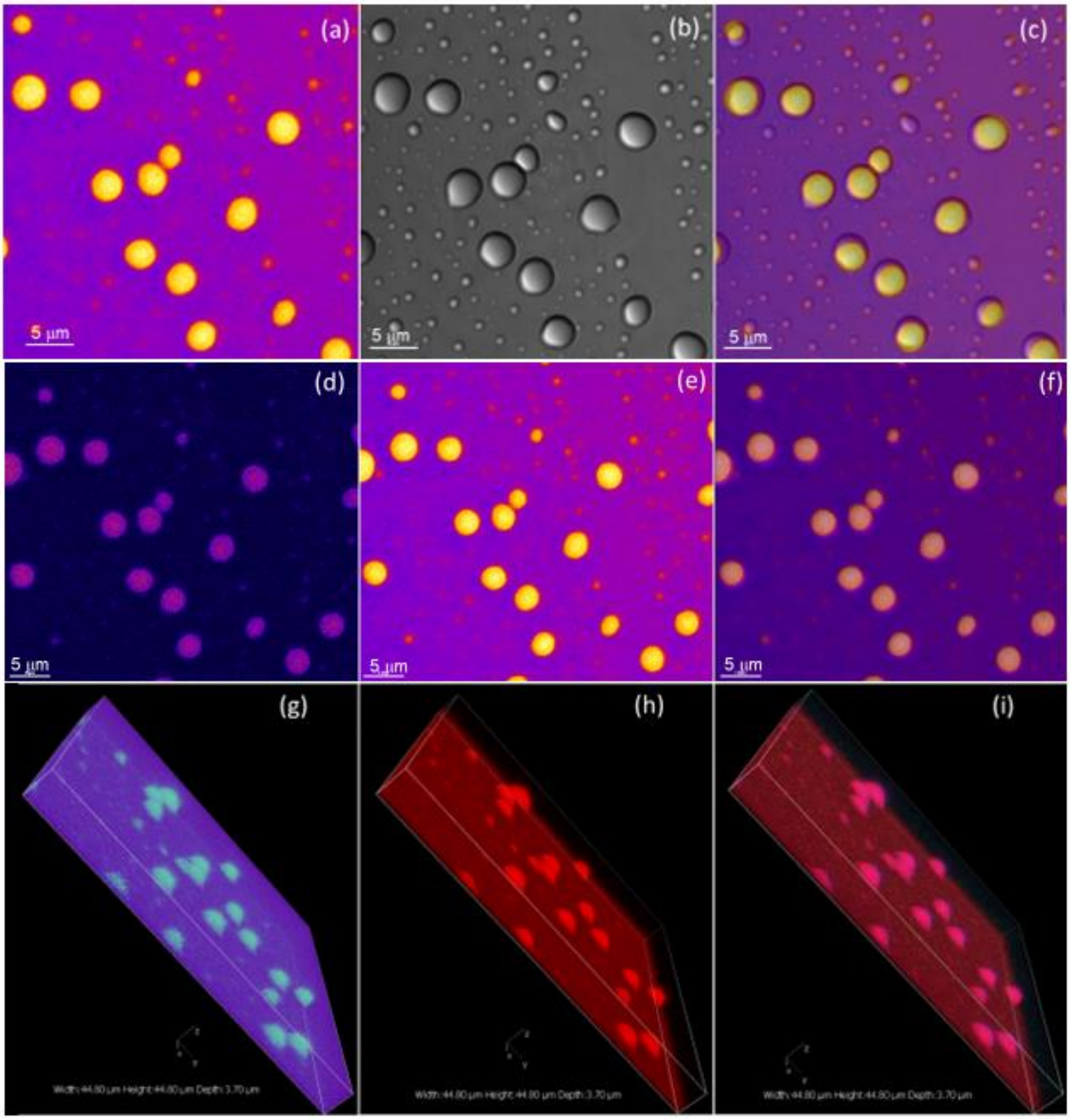

Fig S13. Confocal and transmission microscopy images for coacervates for 3 wt% W_3R_3:2.4wt% ATP charge ratio 1:1. Sample stained with 3×10^{-4} wt% RhoB and 3×10^{-3} wt% quinacrine. (a) Fluorescence of quinacrine, (b) Transmission detector image and (c) overlap of images (a) and (b). (d) Fluorescence of RhoB, (e) quinacrine and (f) overlap of images (d) and (e). 3D images for fluorescence of (g) RhoB, (h) quinacrine and (i) overlap of images (g) and (h).

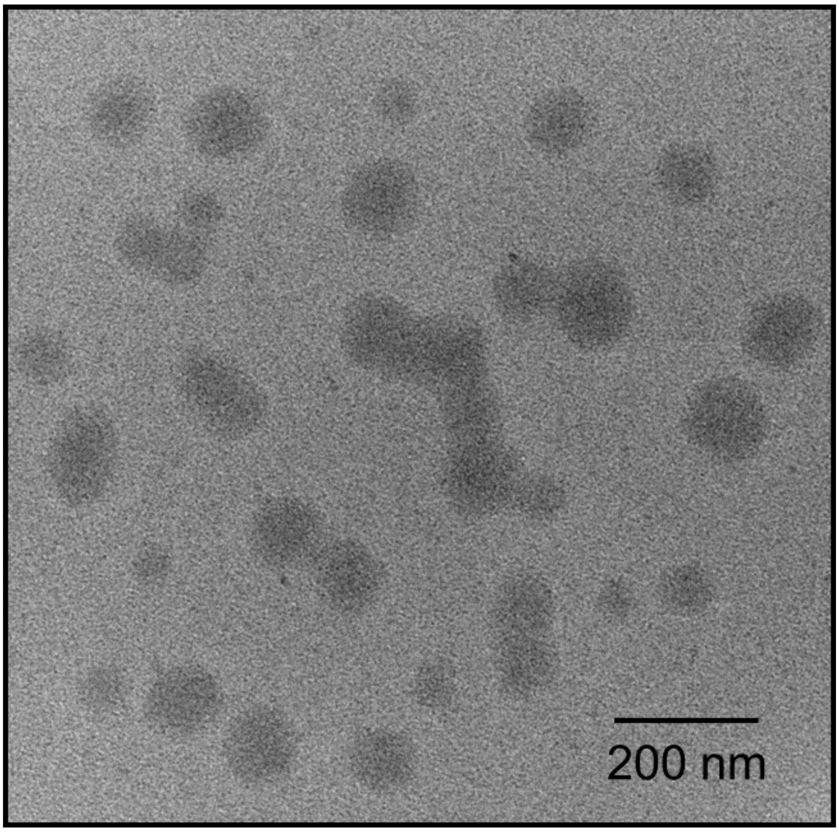

Fig. S14. Cryo-TEM image of coacervate droplets observed for the supernatant of 3 wt% WR:2.3 wt% ATP charge ratio 1:1.

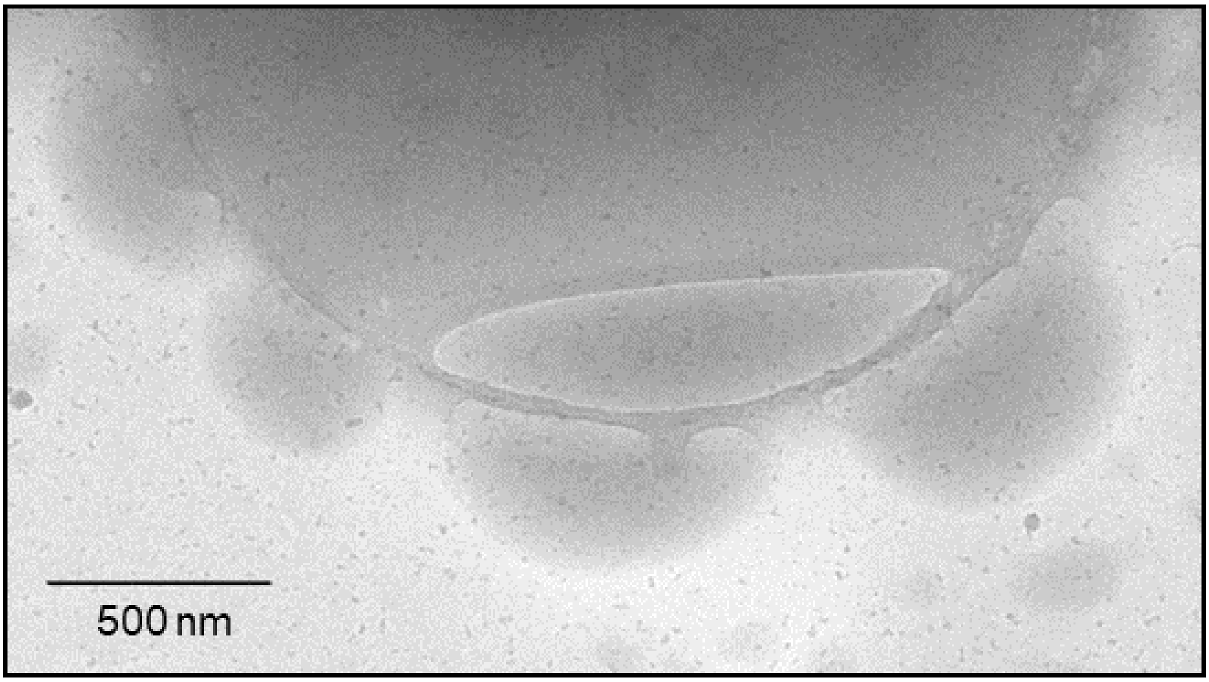

Fig. S15. Cryo-TEM image for coacervate droplets observed for the supernatant of 3 wt% W_2R_2:2.4 wt% ATP charge ratio 1:1.

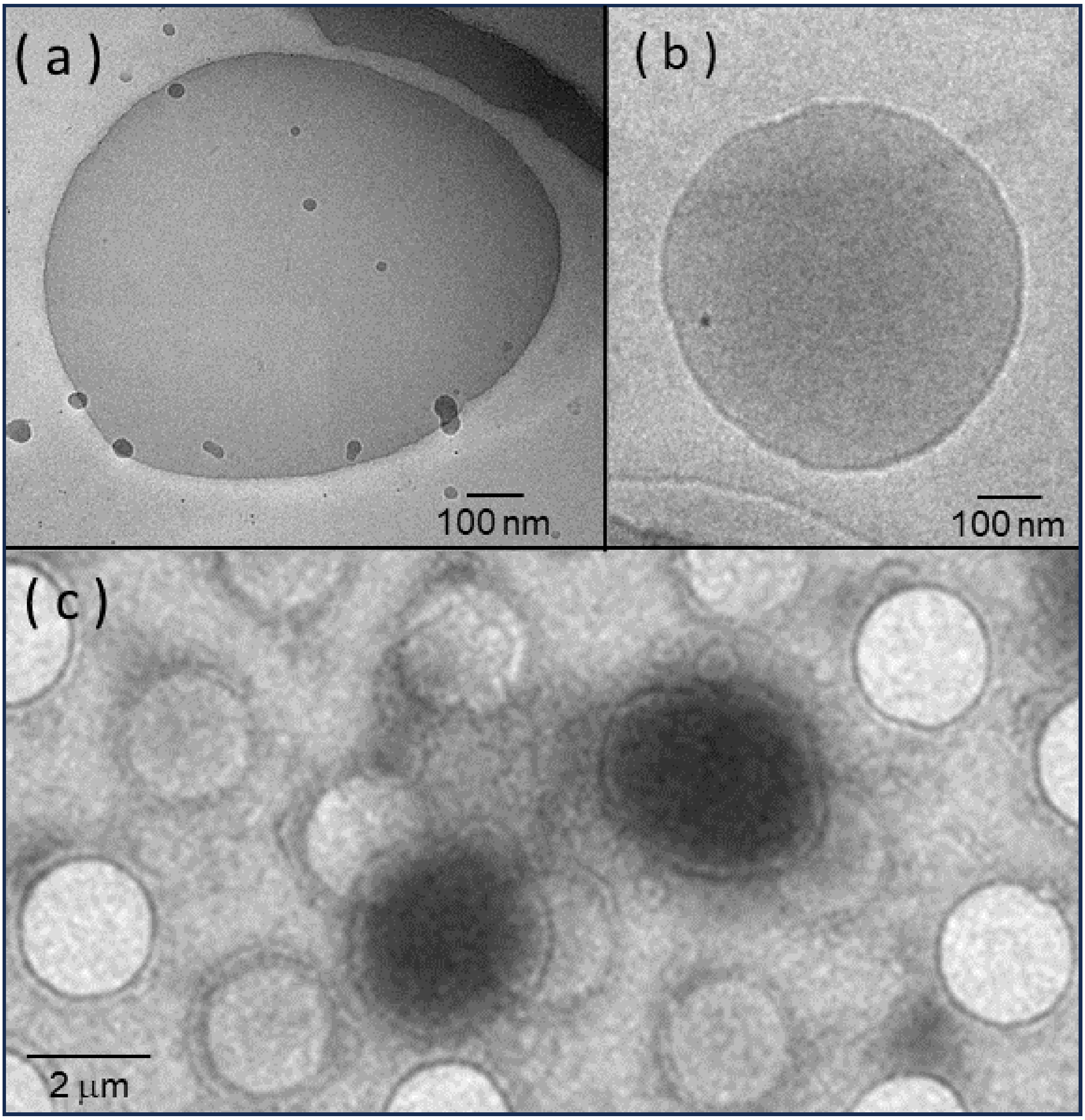

Fig. S16. (a-c) Cryo-TEM images for coacervates observed for the supernatant of 3 wt% W_3R_3:2.4 wt% ATP charge ratio 1:1. The light regions in part (c) are TEM grid holes.

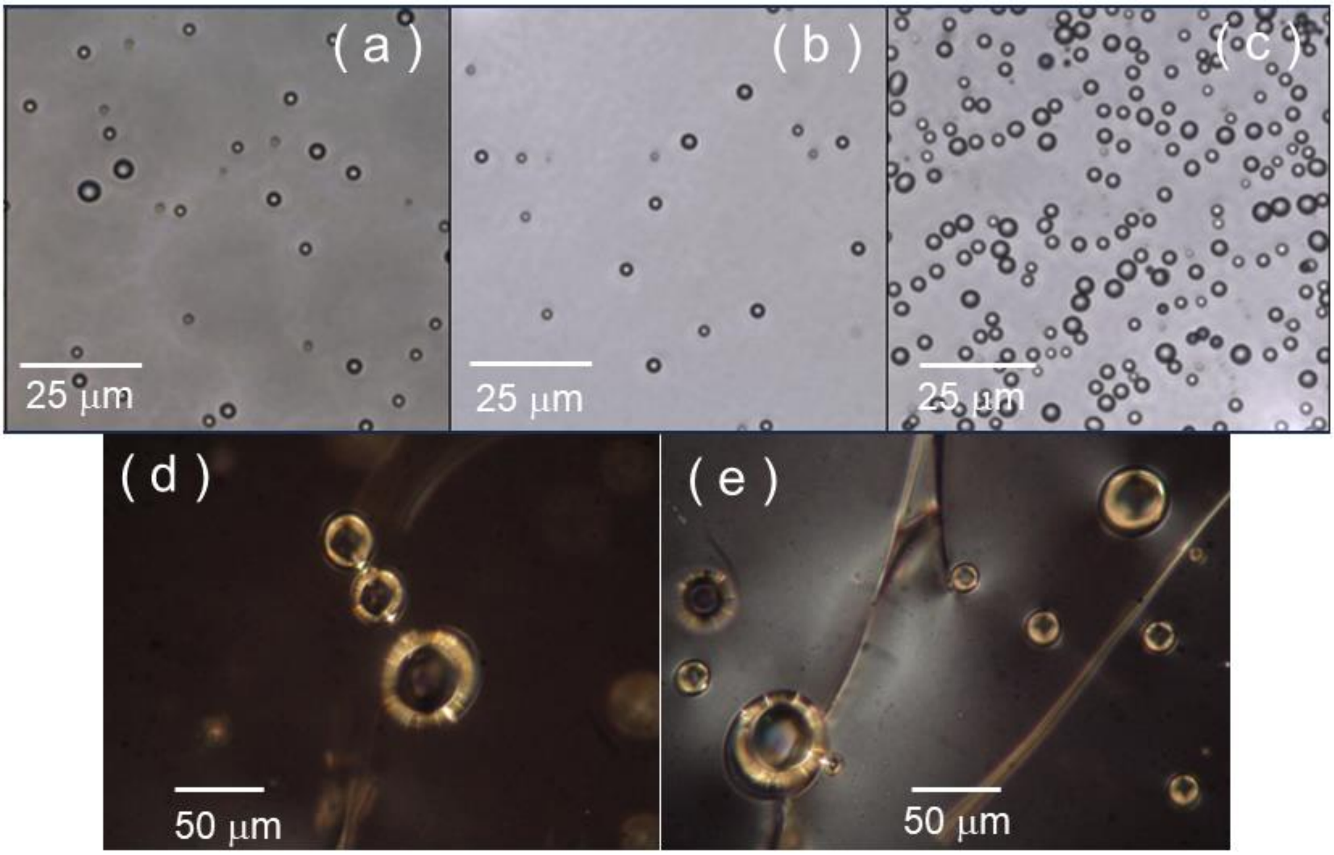

Fig. S17. Images for samples at 1:0.5 peptide:ATP charge ratio. Optical microscope images for (a) 3 wt% WR:1.2wt% ATP. Supernatant for (b) 3 wt% W_2R_2:1.2wt% ATP and (c) 3 wt% W_3R_3:1.2wt% ATP. (d-e) Polarized optical microscopy images for W_2R_2:ATP precipitates from the sample with supernatant imaged in (b).

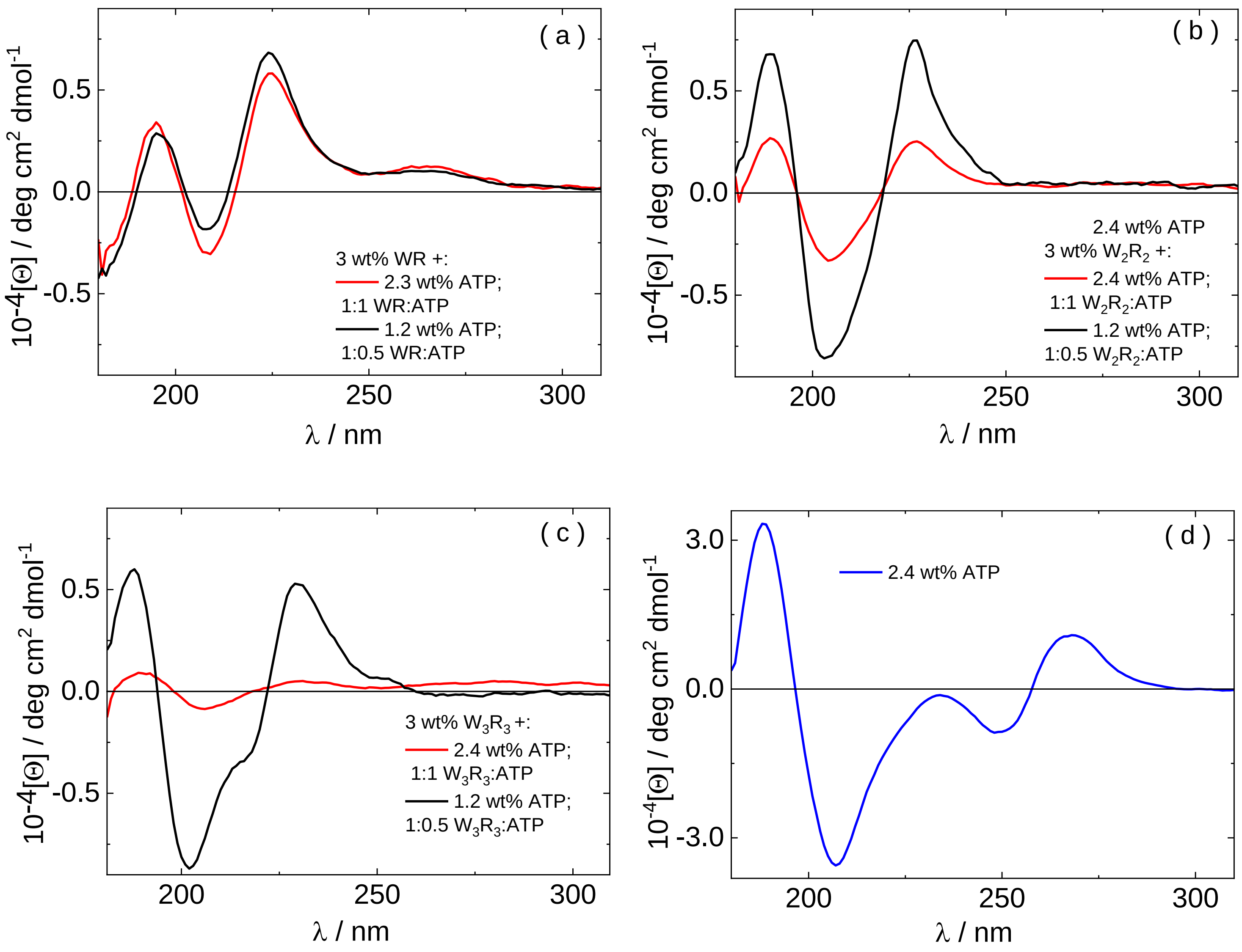

Fig.S18. CD spectra for mixtures with ATP for the mixtures indicated (a) WR: ATP, (b) W_2R_2 ATP, (c) W_3R_3: ATP, (d) ATP.

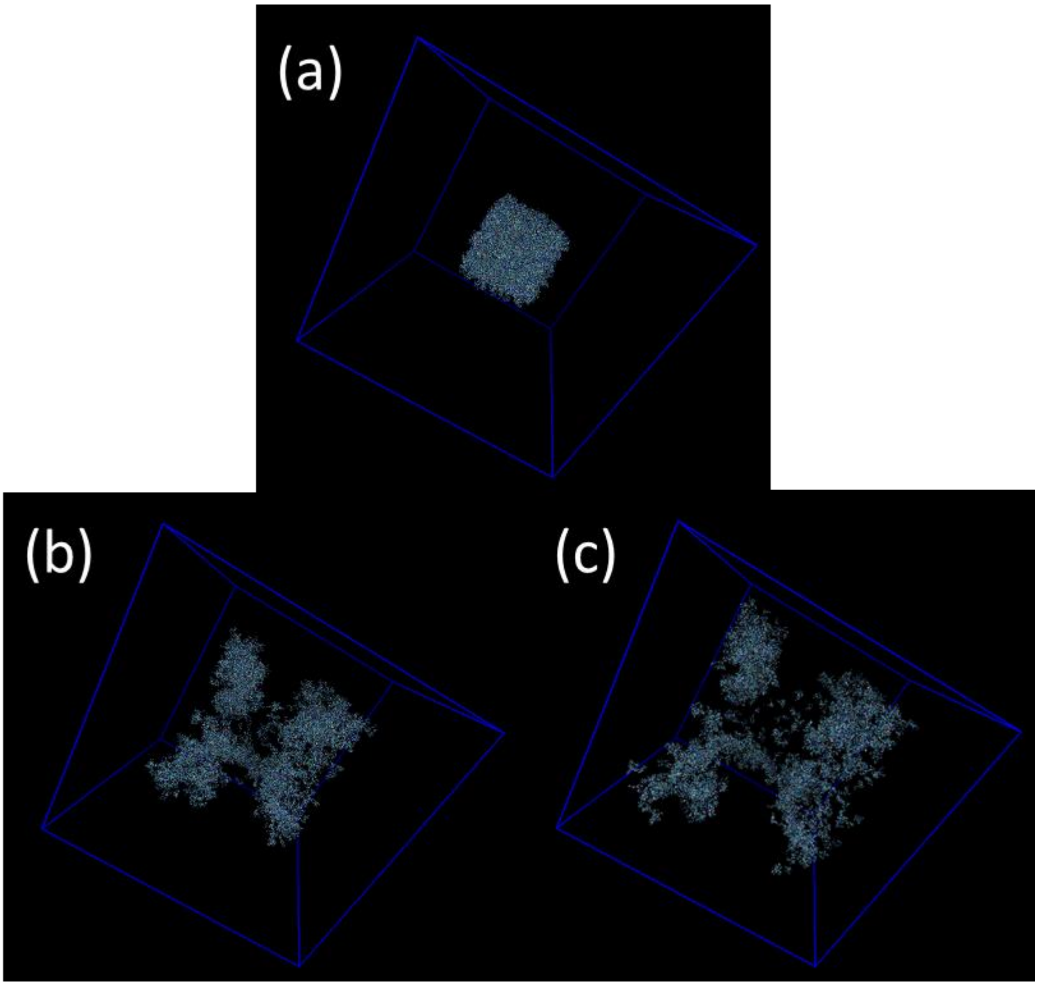

Fig. S19. Frames showing peptide structure evolution of W_2R_2 in water during MD simulation trajectory. (a) Start (t =0), (b) t = 250 ps, (c) t = 1000 ps. Box is the 40 nm simulation box (water molecules not shown).

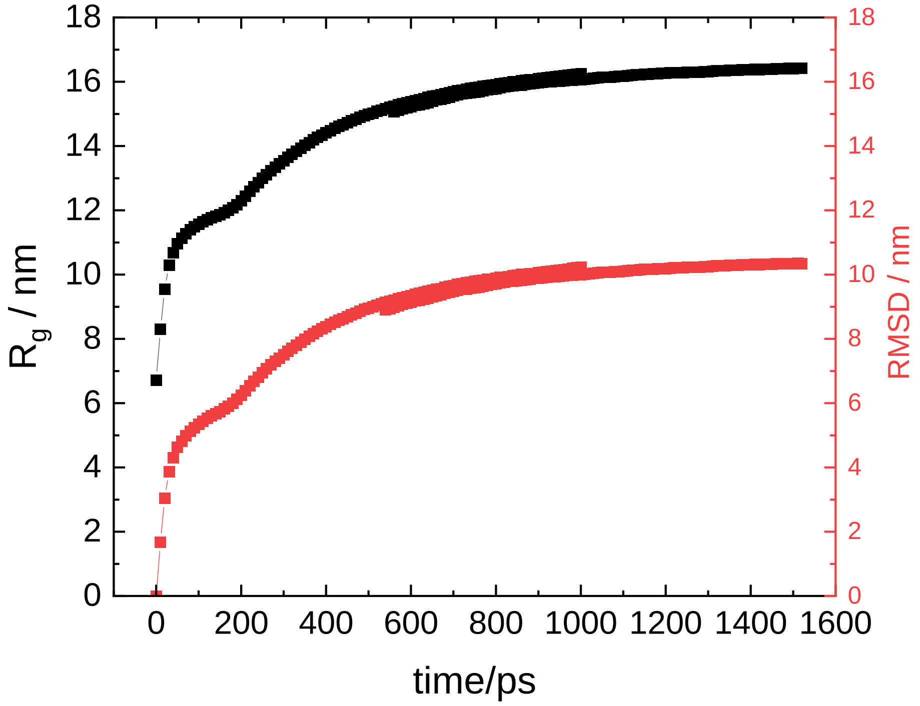

Fig. S20. MD trajectory analysis of all 81,600 W_2R_2 peptide molecules, showing radius of gyration, R_g and root-mean-square deviation (RMSD). Data from initial and extended runs (with overlap).

(a)

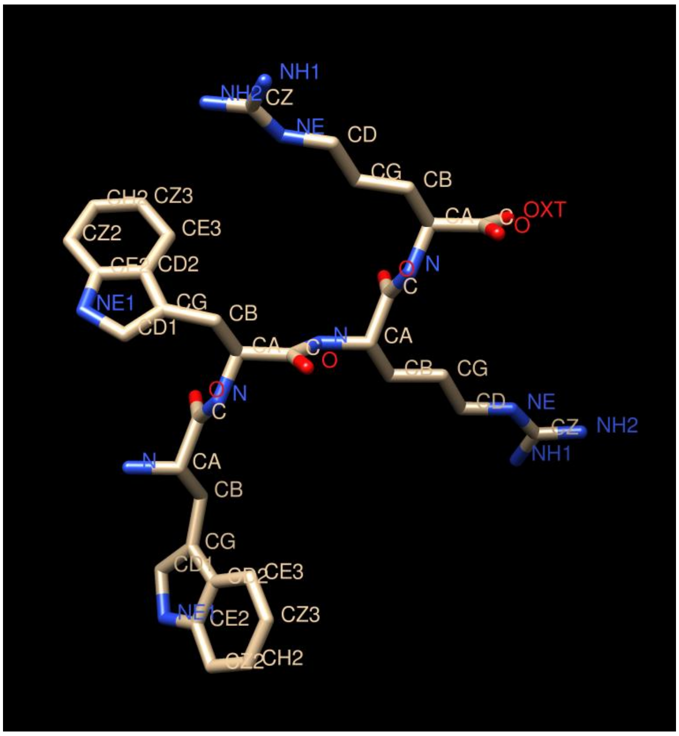
NH1
NH2
CZ
NE
CD
CG
CB
CH2
CZ3
CE3
CZ2
CD2
NE1
CG
CB
CD1
CA
OXT
O
N
C
CA
CB
CG
NE
NH2
NH1
CA
CB
CG
CE3
CE2
CZ3
CH2

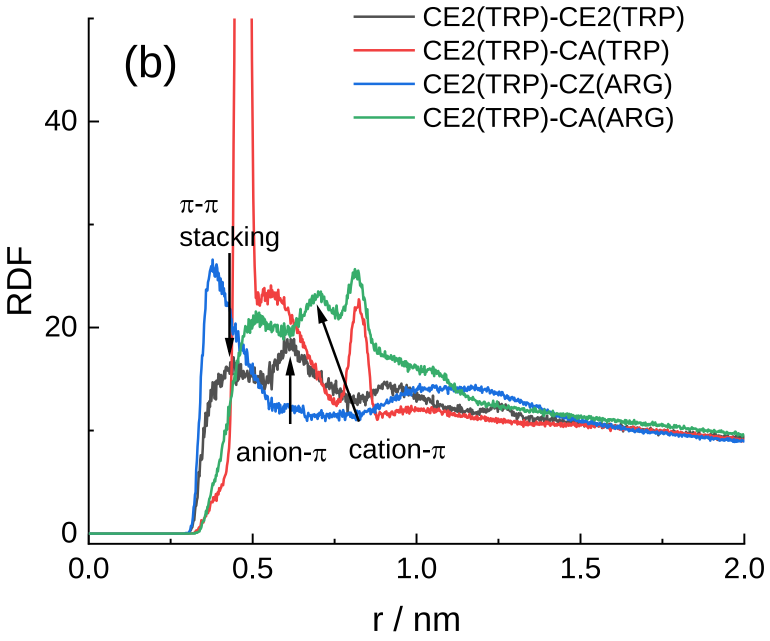
(b)
CE2(TRP)-CE2(TRP)
CE2(TRP)-CA(TRP)
CE2(TRP)-CZ(ARG)
CE2(TRP)-CA(ARG)
π-π
stacking
anion-π
cation-π
RDF
0
20
40
0.0
0.5
1.0
1.5
2.0
r / nm

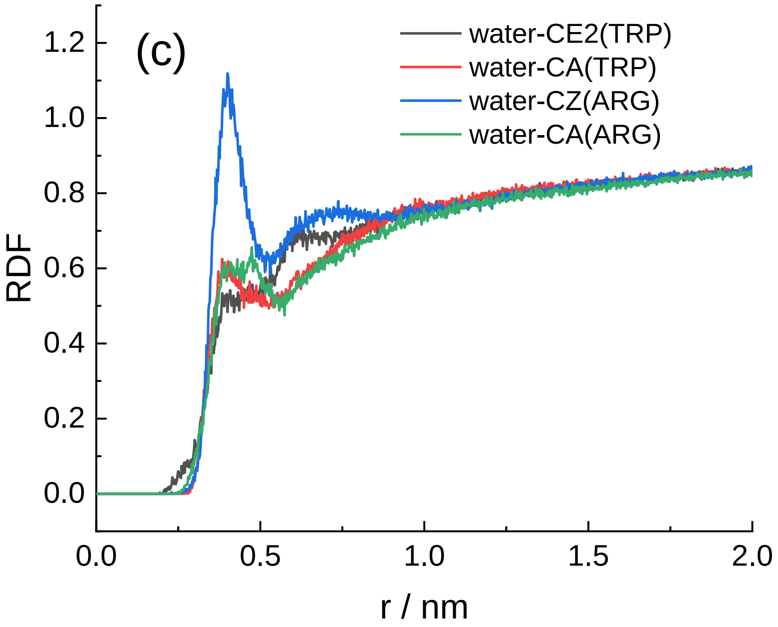

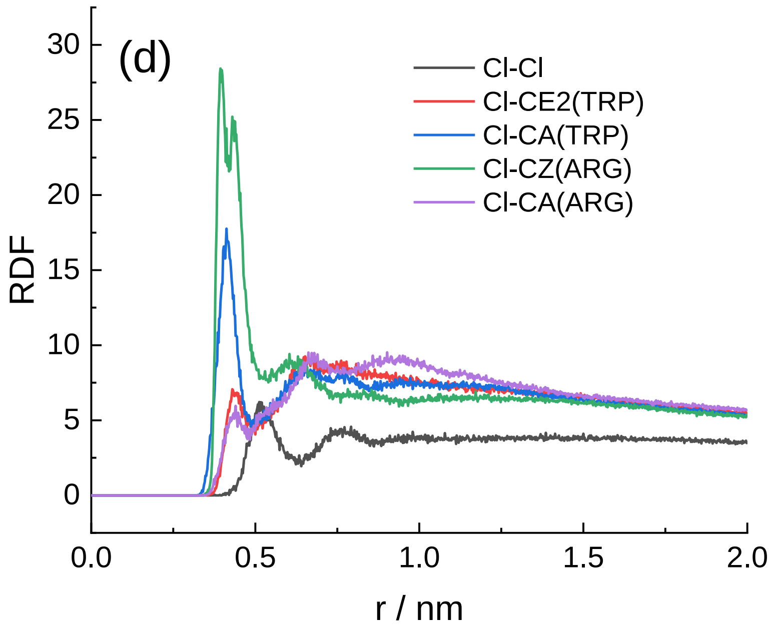

Fig.S21. Radial distribution function (RDF) analysis. (a) Atom labelling, (b) RDFs associated with W aromatic group (CE2 selected), (c) RDFs associated with water molecules, (d) RDFs associated with Cl^{-} counterions.

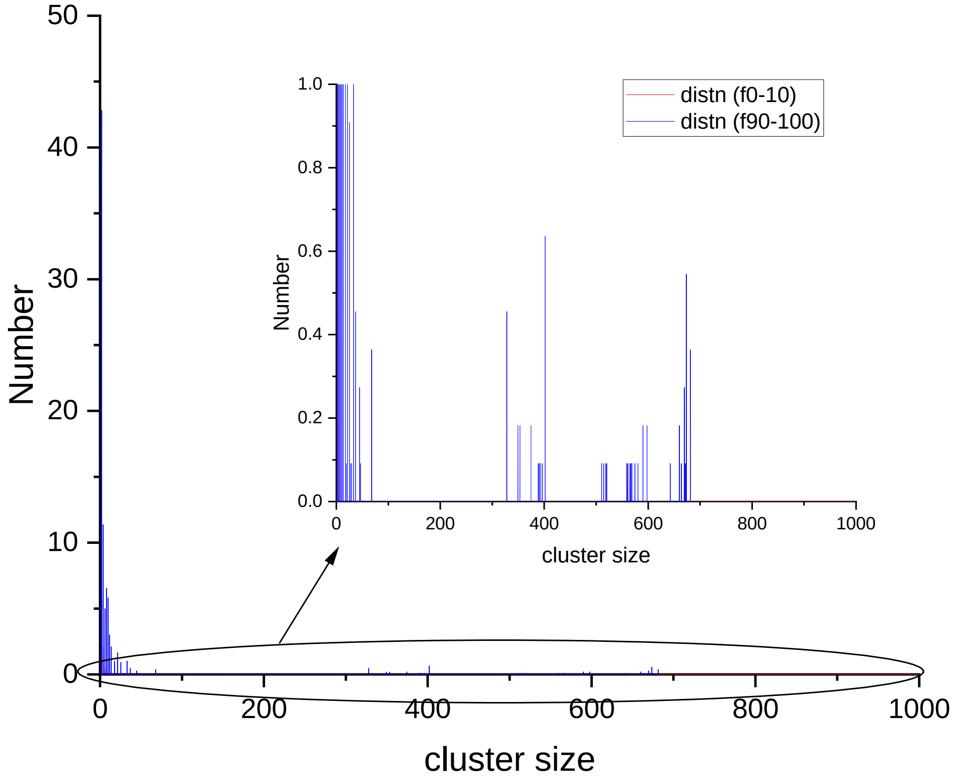

Fig.S22. Showing the development of clusters (molecular cluster size), comparing initial frames (f0 – 10, 0-100 ps) with frames 90-100 (900 – 1000 ps) in the simulation for W_2R_2.

Table S1. Zeta-potential data.

**Sample**	**Coacervation**	**Zeta Potential mV**
1 wt% W_2R_2 in water; pH 2.68	No	11.9±1.4
2.4 wt% W_2R_2; pH 6.6	No	22.1±1.9
2.2 wt% W_2R_2; pH 6.4	No	11.4±1.9
1 wt% W_2R_2; pH 11.3	boundary	10.1±0.1
1 wt% W_2R_2; pH 12.5	Yes	-3.6±0.1
2.6 wt% W_2R_2; pH 12	Yes	-3.1±1.0
1 wt% W_3R_3 in water; pH 2.69	No	17.4±1.5
1 wt% W_3R_3; pH 7.7	No	23.5±2.9
1 wt% W_3R_3; pH 12.5	Yes	1.1±0.2
0.1 wt% W_3R_3; pH 12.1	Yes	6.3±0.5

Table S2. SAXS fitting parameters for data in Fig.3c and SI Fig.S8d. The models used were generalized Gaussian coil (monomers) plus sloping forward scattering for the coacervate (pH 12) samples and generalized Gaussian coil (with structure factor for W_2R_2 and W_3R_3) for the native pH samples. Data fitted using SASfit.[1, 2]

Parameter	W_2R_2 pH 12	W_3R_3 pH 12	WR pH 2.5	W_2R_2 pH 2.5	W_3R_3 pH 2.5
I_0 / cm^{-1}	0.011	0.107	0.0047	0.0070	0.0096
R_g / Å	9.23	18.95	6.02	9.48	9.40
ν	0.173	0.178	0.156	0.165	0.164
I_s / cm^{-1}	1.22×10^{-10}	8.25×10^{-10}	-	-	-
n [a]	4	4	-	-	-
BG / cm^{-1}	4.81×10^{-3}	5.72×10^{-3}	3.42×10^{-3}	6.54×10^{-3}	7.08×10^{-3}
I_G / cm^{-1}	-	-	-	1.22	1.92
q_G /Å^{-1} [a]	-	-	-	0.15	0.15
w_G / [a]	-	-	-	0.1	0.1

Key. Generalized Gaussian coil: I_0, forward scattering; R_g, radius of gyration; v, Flory exponent. **Sloping background**: $I_s q^{-n}$ + BG where BG is constant background. **Structure factor peak**: I_G, peak amplitude; q_G peak centre; w_G peak width. [a] Fixed parameter

Table S3. Renormalized Tryptophan Partial Charges (atom labelling scheme in Fig.S21a)

Group	Amber03 charge	Renormalized Charge
CA	-0.020082	0.062459
HA	0.106629	0.053298
CB	-0.098364	-0.034085
HB1	0.065424	0.048137
HB2	0.065424	0.048137
CG	-0.099797	-0.213891
CD1	-0.174053	-0.102060
HD1	0.170633	0.219396
NE1	-0.298433	-0.377408
HE1	0.322375	0.377656
CE2	0.141523	0.093735
CZ2	-0.210701	-0.200773
HZ2	0.125512	0.153402
CH2	-0.133022	-0.109764
HH2	0.119467	0.144857
CZ3	-0.164054	-0.258511
HZ3	0.119250	0.161000
CE3	-0.153992	-0.082900
HE3	0.123084	0.125881
CD2	0.089641	0.117487

References

1. Bressler, I.; Kohlbrecher, J.; Thünemann, A. F., SASfit: a tool for small-angle scattering data analysis using a library of analytical expressions. *Journal of Applied Crystallography* **2015,** *48*, 1587-1598.
2. Kohlbrecher, J.; Bressler, I., Updates in SASfit for fitting analytical expressions and numerical models to small-angle scattering patterns. *Journal of Applied Crystallography* **2022,** *55*, 1677-1688.
